# Bounded Integer Modeling of Symptom Scales Specific to Lower Urinary Tract Symptoms Secondary to Benign Prostatic Hyperplasia

**DOI:** 10.1208/s12248-021-00568-y

**Published:** 2021-02-25

**Authors:** Yassine Kamal Lyauk, Daniël M. Jonker, Andrew C. Hooker, Trine Meldgaard Lund, Mats O. Karlsson

**Affiliations:** 1 Translational Medicine, Ferring Pharmaceuticals A/S, Kay Fiskers Plads, 11 Copenhagen, Denmark; 2 Department of Drug Design and Pharmacology, University of Copenhagen, Copenhagen, Denmark; 3 Department of Pharmaceutical Biosciences, Uppsala University, Uppsala, Sweden

**Keywords:** BPH, BPH impact index, International Prostate Symptom Score, LUTS, Quality of life

## Abstract

The International Prostate Symptom Score (IPSS), the quality of life (QoL) score, and the benign prostatic hyperplasia impact index (BII) are three different scales commonly used to assess the severity of lower urinary tract symptoms associated with benign prostatic hyperplasia (BPH-LUTS). Based on a phase II clinical trial including 403 patients with moderate to severe BPH-LUTS, the objectives of this study were to (i) develop traditional pharmacometric and bounded integer (BI) models for the IPSS, QoL score, and BII endpoints, respectively; (ii) compare the power and type I error in detecting drug effects of BI modeling with traditional methods through simulation; and (iii) obtain quantitative translation between scores on the three abovementioned scales using a BI modeling framework. All developed models described the data adequately. Pharmacometric modeling using a continuous variable (CV) approach was overall found to be the most robust in terms of type I error and power to detect a drug effect. In most cases, BI modeling showed similar performance to the CV approach, yet severely inflated type I error was generally observed when inter-individual variability (IIV) was incorporated in the BI variance function (g()). BI modeling without IIV in g() showed greater type I error control compared to the ordered categorical approach. Lastly, a multiple-scale BI model was developed and estimated the relationship between scores on the three BPH-LUTS scales with overall low uncertainty. The current study yields greater understanding of the operating characteristics of the novel BI modeling approach and highlights areas potentially requiring further improvement.

**Supplementary Information:**

The online version contains supplementary material available at 10.1208/s12248-021-00568-y.

## Introduction

Benign prostate hyperplasia (BPH) is a common condition in the aging male and is estimated to affect 50% of males by age 60 years and 90% by age 85 years (1,2). The increase in prostatic weight frequently leads to a spectrum of clinical manifestations known as lower urinary tract symptoms (LUTS). The severity of BPH-LUTS is most commonly measured by the International Prostate Symptom Score (IPSS) (also known as the American Urological Association score) (3), which consists of seven questions describing the severity of symptoms. These comprise the feeling of incomplete bladder emptying, frequency of urination, intermittency during urination, the urgency to urinate, weakness of the urinary stream, straining during urination, and nocturia. Each IPSS question can be scored from 0 to 5, resulting in a summary IPSS that may range from 0 to 35. The IPSS is commonly used as a primary efficacy endpoint in BPH-LUTS clinical trials and is considered the gold standard diagnostic tool in the clinic (4,5). In addition to the IPSS, two additional endpoints for assessing BPH-LUTS are regularly implemented in clinical trials: the quality of life (QoL) score (6) and the BPH impact index (BII) (7). The QoL question evaluates a patient’s perception of his ability to tolerate his current level of BPH-LUTS for the rest of his life. It is rated between 0 and 6, with 0 corresponding to feeling “delighted” regarding this outlook and 6 corresponding to feeling “terrible.” Research has previously pointed towards the utility of the QoL score to diagnose BPH-LUTS severity and its significant correlation to the IPSS (6). The BII questionnaire consists of four questions that respectively aim to assess the impact of BPH-LUTS on a patient’s level of physical discomfort, degree of worrying, general inconvenience caused by urinary problems, and impediment of desired activity. A summary BII score between 0 and 13 is possible. Significant correlation with the IPSS and QoL score has been established (8), as well as clinical significance thresholds of changes in the IPSS and the BII (9). However, the direct connection between observed scores on each of the three abovementioned BPH-LUTS scales has to our knowledge not been investigated. Given the use of different rating scales in the clinic and in clinical trials within BPH-LUTS, such knowledge may allow for informed translation between scales. This may be important in the context of clinical research as well as for bridging between clinical trials.

Traditionally, model-based analysis of rating scales with more than 10 categories treats the data as a continuous variable (CV) (10), which violates the inherent discrete nature of the data. On the other hand, using an ordered categorical (OC) approach may require too many parameters and does not allow for prediction of categories that are not observed in the data. Recently, bounded integer (BI) modeling was presented as a method for describing data originating from scales (11). The BI approach often showed lower Akaike information criterion values compared to the CV and OC approaches in the analysis of several different scales (11), indicating that it may be a promising method for longitudinal analysis of clinical trials employing scale endpoints. However, the power to detect a drug effect and corresponding type I error of the BI modeling approach has not yet been examined. This knowledge may contribute to solidifying the utility of BI modeling in comparison to traditionally used methods. The objectives of the current work were hence to (i) develop BI models for the IPSS, QoL score, and BII BPH-LUTS scales based on data from a phase II double-blind parallel-group clinical trial; (ii) compare the power and type I error in detecting a drug effect of the developed BI models with traditional continuous variable and/or ordered categorical modeling models, respectively; and (iii) develop a BI model regarding trial data from the three BPH-LUTS scales simultaneously to establish the connection between scores on the different scales.

## Methods

### Data

Ferring Pharmaceuticals A/S trial CS36 (NCT00947882) was a phase II double-blind, parallel-group, dose-finding study evaluating the efficacy and safety of a single subcutaneous injection of the GnRH antagonist degarelix over 6 months. Four hundred and three patients with moderate to severe BPH-LUTS were randomized to placebo, 10-, 20-, or 30-mg degarelix 40 mg/mL solution. For trial inclusion, all patients were required to have an IPSS ≥ 13 and a QoL ≥ 3 at screening 2 weeks before dosing at the baseline visit. Each patient had seven scheduled visits post-baseline (14, 30, 61, 91, 121, 152, and 182 days post-dose). The IPSS and QoL score was measured at all eight trial visits, while the BII was measured at baseline, 91 days post-dose, and 182 days post-dose. CS36 was conducted in accordance with the Declaration of Helsinki and Good Clinical Practice guidelines. Further details regarding the CS36 trial have been presented previously elsewhere (12,13).

### Modeling methodology

#### Model development

##### Continuous variable modeling

A continuous variable (CV) longitudinal IPSS model previously presented elsewhere (12) was used as basis for comparison with the BI approach in the current study. The CV IPSS model was developed on the current CS36 data and was modified to contain no covariates or IIV distribution transformations. The longitudinal trajectory of the IPSS was described according to

$${\displaystyle \begin{array}{c}\begin{array}{c} IPSS= Baseline+ Placebo(t)+ Drug\\ {} Placebo(t)= Pmax\left(1-{e}^{-\frac{\ln (2)}{Tprog}\ast Time}\right)+ Drift\ast Time\end{array}\\ {} Drug=0\kern0.75em if\ Dose=0\kern0.5em or\ Time=0\\ {} Drug=\theta \kern0.5em if\ Dose>0\kern0.5em \& \kern0.5em Time>0\end{array}}$$

with *Baseline* representing the baseline IPSS, *Pmax* the maximal placebo effect, *Tprog* the half-life to reach *Pmax*, *Drift* describing relapse or continued remission over time, and *Drug* the offset drug effect of degarelix. IIV was included for *Baseline* and *Tprog* assuming a lognormal distribution while IIV for *Pmax* and *Drift*, respectively, were assumed normally distributed. A combined residual error model was used.

For the modeling of BII data, time-varying placebo effect models such as the linear, bi-linear, power, exponential, Weibull, and inverse Bateman structural placebo models were tested, as well as a time-independent placebo effect model (intercept), and combinations thereof (slope-intercept). Offset, linear, and onset drug effect models were examined to describe degarelix treatment effect on the BII scale.

##### Ordered categorical modeling

A rule of thumb is to use an ordered categorical (OC) approach when there are less than ten categories (10). As the QoL score consists of seven categories, an OC proportional odds (PO) model (14) was used to model these data. The BII total score contains fourteen discrete response values, all of which apart from the extreme values can be arrived at through many different combinations of responses. However, it may be viewed by some modelers as an ordered categorical variable. An OC PO model was therefore also applied to the total BII score in the current work.

In the OC PO model, the logit of the probability of an observation *Y* being equal or greater than a score *j* in the *i*th individual at time *t* is given by

$${\displaystyle \begin{array}{c} logit\left[P\left({Y}_{it}\ge j|{\eta}_i\right)\right]={f}_j+{\eta}_i\kern1.25em where\ j=1,\dots, X\\ {}\left( with\ X=6\ for\ the\ QoL\ score\ and\ X=13\ for\ the\ BII\right)\\ {}\ giving\ P\left({Y}_{it}\ge j|{\eta}_i\right)=\frac{e^{f_j+{\eta}_i}}{1+\kern0.5em {e}^{f_j+{\eta}_i}}\end{array}}$$

where *f*_j_ is a function of the baseline probability of each score (*α*_*m*_) and the effect of predictors (in the current work, the time-dependent placebo, and drug effect, respectively):

$${f}_j=\sum \limits_{m=1}^j{\alpha}_m+ Placebo(t)+ Drug(t)$$

and η_i_ is the inter-individual variability (IIV), with mean zero and variance ω^2^. Similar placebo and drug effect models to those mentioned for CV BII model development were investigated in OC model development for the QoL and BII scales, respectively. Lastly, the probability of observing a particular score is given by

$${\displaystyle \begin{array}{c}P\left({Y}_{in}=0\right)=P\left({Y}_{in}\ge 0\right)-P\left({Y}_{in}\ge 1\right)=1-P\left({Y}_{in}\ge 1\right)\\ {}P\left({Y}_{in}=1\right)=P\left({Y}_{in}\ge 1\right)-P\left({Y}_{in}\ge 2\right)\\ {}P\left({Y}_{in}=X\right)=P\left({Y}_{in}\ge X\right)\end{array}}$$

##### Bounded integer modeling

The methodology has been described in detail elsewhere (11). Briefly, given a scale of *n* categories, the probits (Z_*1/n*_ to Z_*(n-1)/n*_) are first calculated to specify *n-1* cut-offs, which border *n* equally-sized areas under a standard normal distribution N(0,1). For the IPSS, 35 cut-offs were used, six cut-offs were used for the QoL, and 13 cut-offs were used for the BII. Subsequently, in conjunction with the probits, the probability of each category is determined through a function describing a normal distribution of fixed (θ) and random effects (η_i_), time, and covariates *f(θ, η*_*i,f*_*, t, X*_*i,f*_)*,* along with a variance function *g(σ, η*_*i,g*_*, t, X*_*i,g*_*)*, i.e., N(*f(θ, η*_*i,f*_*, t, X*_*i,f*_*)* and *g(σ, η*_*i,g*_*, t, X*_*i,g*_*))*. The probability for the *k*th category, *P*_*i,j*_*(k),* is defined as

$${P}_{i,j}(k)=\varPhi \left(\frac{Z_{\frac{k}{n}}-f\left(\theta, {\eta}_{i,f},t,{X}_{i,f}\right)}{g\left(\sigma, {\eta}_{i,g},t,{X}_{i,g}\right)}\right)-\varPhi \left(\frac{Z_{\frac{k-1}{n}}-f\left(\theta, {\eta}_{i,f},t,{X}_{i,f}\right)}{g\left(\sigma, {\eta}_{i,g},t,{X}_{i,g}\right)}\right)$$

with Φ being the cumulative distribution of the normal distribution function, i.e., the area under the latent function curve within the cut-off interval. For the first category (*k = 1*):

$${P}_{i,j}(1)=\varPhi \left(\frac{Z_{\frac{1}{n}}-f\left(\theta, {\eta}_{i,f},t,{X}_{i,f}\right)}{g\left(\sigma, {\eta}_{i,g},t,{X}_{i,g}\right)}\right)$$

representing the cumulative distribution function in the interval [-∞, Z_*1/n*_], and for the last category (*k = n*):

$${P}_{i,j}(n)=1-\varPhi \left(\frac{Z_{\frac{n-1}{n}}-f\left(\theta, {\eta}_{i,f},t,{X}_{i,f}\right)}{g\left(\sigma, {\eta}_{i,g},t,{X}_{i,g}\right)}\right)$$

representing the cumulative distribution in the interval [Z(n-1)/n, ∞].

Similar placebo and drug effect models to those described within CV BII model development were tested on the latent probit scale. Moreover, the addition of a *Drift* parameter similar to what was incorporated in the CV IPSS model was also examined. BI models with and without inter-individual variability (IIV) in the BI variance function, g(), were developed for each scale to precisely assess potential sources of variation in performance. Including an IIV term in the BI variance function allows the scoring consistency to vary between subjects and has previously been shown to reduce the Akaike information criterion substantially (11). In all of the developed BI models that included IIV in g(), a lognormal distribution was specified for this IIV term as described in Wellhagen *et al.* (11).

##### Joint bounded integer modeling of multiple scales

Due to its utilization of a latent scale, a multivariate approach can be implemented under the BI framework, which regards multiple scale measures describing the same disease simultaneously (15). In the current analysis, a joint BI model describing changes in IPSS, QoL, and BII over time in individual patients was developed. The IPSS cut-offs (Z_*1/36*_ to Z_*35/36*_) were specified as the reference (i.e., identical to the probits in the BI model considering only the IPSS scale), while the cut-offs for the QoL and the BII scores, respectively, were estimated on this same latent scale. The relationship between scores from each scale could thereby be established. Given that the IPSS was used as the reference scale in the joint BI model, the longitudinal model was developed according to the trajectory of the IPSS. The same longitudinal model described the longitudinal trajectory of probabilities for the QoL score and the BII. Differences in scale measurement frequency over the trial period is likely to influence the consistency in patients’ scores, and therefore, incorporation of a separate g() variance function was investigated for the BII scale in the joint BI model: BII measurements were only available at three visits in the CS36 trial period compared to eight measurements over the trial period for the IPSS and the QoL score.

#### Model selection and evaluation

For nested models, the difference in objective function value (OFV) corresponding to a significance level of 0.05 was considered statistically significant assuming a χ^2^ distribution. For non-nested models, the difference in Akaike information criterion (AIC) was used. AIC was computed as the objective function value (OFV) plus two times the number of model parameters. Model stability based on the convergence of minimization and covariance steps, parameter precision assessed through NONMEM’s relative standard error estimate, and graphical diagnostics was also considered during model selection. Visual predictive checks (VPCs) were used to assess the adequacy of the model characterization of the observed longitudinal data on each scale.

### Power and type I error calculation

The analysis of power and type I error considered multiple simulation scenarios from different types of models using BPH-LUTS scales that differ in the number of possible scores as case studies. The latter allowed for comparison of the BI model with different types of reference models (CV and/or OC). Simulating data from only type of model would bias the analysis and therefore the current work sought to gain an overall understanding of the operating characteristics of the BI approach by testing its performance under different conditions. A stochastic simulation and estimation (SSE) procedure with 500 trial replicates at different sample sizes was used to assess the power to detect a drug effect of the developed models. In the SSE, a drop in OFV of 3.841 (*p* = 0.05 assuming a χ^2^ distribution) was used as the threshold to establish statistical significance of the drug effect between the reduced (without a drug effect parameter) and full models (with a drug effect parameter). All simulated trials had the same allocation ratio of patients in the placebo and treatment arms as in the CS36 clinical trial. No dropout was simulated for simplicity purposes. For the comparison of power of the BI IPSS model and the CV IPSS model, a pharmacometric item response theory (IRT) model (12) that was previously developed on the same CS36 data set as in the current study was used as the simulation model. Simulated total IPSS responses were hence obtained from the sum of simulated item-level IPSS responses. For power estimation of models regarding the QoL score and the BII, respectively, the simulation model was a previously developed integrated IRT model (13), which was also developed on the same data used in the current work. In the investigation of power to detect of a drug effect of the developed models within each BPH-LUTS scale, pharmacometric IRT models, which describe the respective observed longitudinal trajectories of the IPSS, QoL score, and BII in the CS36 trial adequately (12,13), were chosen as the simulation model in the main investigational scenario to minimize simulation model bias and generate integer scores as would be observed in, e.g., an actual clinical trial within BPH-LUTS. Additionally, SSE procedures were also performed using each of the different developed models that inherently respect the integer nature of the data (i.e., OC and BI models) as the simulation model. The type I error of the models in detecting a drug effect was investigated in an identical fashion to power, except for the simulation models containing no drug effect.

### Software

Modeling was carried out using the Laplace estimation method in NONMEM version 7.4.3 and Perl-Speaks-NONMEM (PsN) (16) version 4.9.0. Laplacian estimation with interaction was used for the continuous variable models. R version 3.6.0 and the xpose4 package (16) were used for the post-processing of results and graphics.

## Results

The baseline CS36 trial characteristics and the mean time course of each BPH-LUTS scale have been presented elsewhere (12,13). In summary, each of the four trial arms showed a marked mean decrease from baseline for all three BPH-LUTS scales. Furthermore, by visual inspection of the mean data from the three degarelix treatment arms, no dose-response relationship was evident on any of the symptom scales. In total, 3117 IPSS, 3119 QoL scores, and 1116 BII responses from 403 patients were available for analysis.

### Model development

#### International Prostate Symptom Score

##### Reference model

The parameter estimates along with their relative standard errors in the CV IPSS model (*IPSS-A*) are shown in Table I. Implementing dose-response or exposure-response models (linear and Emax) did not decrease the OFV significantly. A VPC of the CV IPSS model indicated adequate description of the data and is shown in the Supplemental Material.

**Table I:** Parameter estimates in the continuous variable and bounded integer (BI) models of the International Prostate Symptom Score. g() is the variance function in the bounded integer (BI) model

Parameter	Continuous variable (*IPSS-A*)	RSE	BI model without random effect in g() (*IPSS-B*)	RSE	BI model with random effect in g() with *Drift* (*IPSS-C*)	RSE	BI model with random effect in g() without *Drift* (*IPSS-D*)	RSE
Baseline	19	1.2%	0.152	12.6%	0.108	18.1%	0.109	3%
Asymptote	-3.82	11.7%	-0.287	13.7%	-0.29	10.6%	-0.284	24.7%
Progression half-life	13.6	22.4%	14.3	35.7%	17.8	18.5%	19	6.6%
Drug effect	-2.08	22.5%	-0.173	27.7%	-0.13	19.8%	-0.145	9.9%
Standard deviation BI (g())	-	-	0.208	3.1%	0.163	5.9%	0.162	10.1%
IIV								
Baseline	19%	4.8%	30.7%	6.6%	27.9%	7.8%	27.8%	7.6%
Asymptote	119.2%	17.9%	37.4%	17.3%	34.6%	9.7%	38.5%	5.6%
Progression half-life	47.6%	15.6%	47.9%	17.6%	72%	12%	70.9%	10.9%
Drift	2.6%	9.4%	0.2%	9.3%	0.0002%	76.8%	-	-
Asymptote-Drift correlation	39.3%	19.8%	-39.0%	28.2%	99%	34.4%	-	-
Standard deviation BI (g())	-	-	-	-	63.7%	13%	64.3%	11.6%
Residual error								
Proportional	11.7%	11.4%	-	-	-	-	-	-
Additive	187.6%	8.6%	-	-	-	-	-	-

*IIV* Inter-individual variability, *RSE* relative standard error

##### Bounded integer models

A BI model without inter-individual variability (IIV) in the variance function, g(), was first developed (model *IPSS-B*). In this model, the longitudinal trajectory of the IPSS was described by the same structural model as in the CV approach (model *IPSS-A*). In Table I, the *Baseline* parameter refers to the *z*-score from a normal distribution, and consequently the placebo and drug effect represent changes on this latent scale. Normally distributed IIV was included for the *Baseline*, *Pmax*, and *Drift* parameters, while lognormal IIV was specified for *Tprog*. Incorporation of an offset drug effect resulted in an OFV drop of 22.2. In the second BI model (*IPSS-C*), IIV was included for the g() function assuming a lognormal distribution, and this yielded a drop in OFV of 393.6 compared to *IPSS-B*. However, in the presence of the g() IIV parameter, the *Drift* parameter was no longer significant and was removed in a fourth model (*IPSS-D*). Incorporation of an offset drug effect resulted in an OFV reduction of 38.2 and 51.9 in *IPSS-C* and *IPSS-D*, respectively. Parameter estimates for the three BI IPSS models are shown in Table I. Similar to the CV IPSS model (*IPSS-A*), no dose-response or exposure-response relationship was found to be significant. VPCs for each of the three BI IPSS models showed adequate description of the data and are presented in the Supplemental Material, along with the NONMEM code used for estimation and simulation.

#### Quality of life score

##### Reference model

An OC proportional odds model was developed (*QoL-A*) where the longitudinal change in logit probability of observing a QoL score, *Y*, was described by

$$logit\left(P\left(Y\ge X\right)\right)={B}_x+ Pmax\left(1-{e}^{-\frac{\ln (2)}{Tprog}\ast Time}\right)+{\eta}_i$$

where X = 1,..,6, B_x_ is the baseline logit probability, *Pmax* is the maximal placebo effect, *Tprog* is the half-life to reach *Pmax*, and η_i_ is the IIV in logit baseline probability. Inclusion of a drug effect parameter did not result in significant OFV reduction. Parameter estimates for the proportional odds model for the QoL score are shown in Table II. A VPC for the OC QoL model (*QoL-A*) is shown in the Supplemental Material, indicating good fit to the data.

**Table II:** Parameter estimates in the ordered categorical proportional odds model and the bounded integer (BI) models of the quality of life score. g() is the variance function in the bounded integer (BI) model

Parameter	Ordered categorical (*QoL-A*)	RSE	BI model without random effect in g() (*QoL-B*)	RSE	BI model with random effect in g() (*QoL-C*)	RSE
B1	10.4	4.1%	-	-	-	-
B2	-3.42	8.9%	-	-	-	-
B3	-2.73	5.7%	-	-	-	-
B4	-2.93	5.0%	-	-	-	-
B5	-2.49	5.9%	-	-	-	-
B6	-3.03	8.3%	-	-	-	-
Baseline	-	-	0.402	5.4%	0.393	67.70%
Asymptote	-2.95	5.4%	-0.426	7.0%	-0.368	54.90%
Progression half-life	15	9.9%	17.1	12.0%	13.5	9.60%
Standard deviation BI (g())	-	-	0.199	5%	0.108	8.50%
IIV						
Baseline	286.7%	5.7%	34.9%	6.6%	34.6%	14.9%
Asymptote	-	-	39.9%	17.5%	33.5%	11.8%
Progression half-life	-	-	39.5%	111.2%	20%	33.5%
Drift	-	-	0.2%	43.6%	0.2%	43.6%
Standard deviation BI (g())	-	-	-	-	92.7%	5.3%
Asymptote-Drift correlation	-	-	-36.7%	36.7%	-28.9%	21.3%

B_m_ is the baseline logit probability of a QoL score ≥ m (where m = 1,..,6). *IIV* Inter-individual variability, *RSE* relative standard error

##### Bounded integer models

First, a BI model without IIV in g() was developed (*QoL-B*). A longitudinal model similar to the BI IPSS model without IIV in g() (*IPSS-B*) described the data. Next, in the second BI QoL model (*QoL-C*), IIV was included in the g() function, yielding an OFV drop of 590.5 points. No significant drug effect was identified in either BI QoL model. Overall low parameter uncertainty (Table II) and adequate fit to the data as evidenced by VPCs (Supplemental Material**)** was observed.

#### Benign prostatic hyperplasia impact index

##### Reference models

One CV (model *BII-A*) and one OC model (*BII-B*) were developed to describe the BII data. In the BII CV model (*BII-A*), the longitudinal model was specified by

$${\displaystyle \begin{array}{c}\begin{array}{c} BII= Baseline+{Placebo}_{Int}+ Drug\\ {} if\ Time=0\ then\ Placebo=0\ else\ Placebo={\theta}_x\end{array}\\ {} Drug=0\kern0.75em if\ Dose=0\kern0.5em or\ Time=0\\ {}\ Drug={\theta}_y\ if\ Dose>0 \& amp; Time>0\end{array}}$$

where *Baseline* is the baseline BII, *Placebo*_*Int*_ is the intercept placebo effect model, and *Drug* is the offset degarelix effect. IIV was included for *Baseline* as well as *Placebo*_*Int*_ assuming a normal distribution. An additive model best described the residual error. Incorporation of an offset drug effect decreased the OFV by 4.6. Incorporating of dose-response or exposure-response models did not yield a significant decrease in OFV. A VPC for the CV BII model (*BII-A*) is presented in the Supplemental Material, indicating adequate fit of the model to the observed data.

In the OC model (*BII-B*), the same type of longitudinal model as in the BII CV model (*BII-A*) described the time course of the logit probability for each score. An additive IIV term was added to the baseline logit probability. Incorporation of an offset drug effect yielded an OFV reduction of 7.1 points. A categorical VPC shows the adequate fit of the model to the data (Supplemental Material), and parameter estimates for both the CV (*BII-A*) and OC (*BII-B*) model are shown in Table III.

**Table III:** Parameter estimates in the continuous variable, ordered categorical (OC), and bounded integer (BI) models of the benign prostatic hyperplasia impact index

Parameter	Continuous variable (*BII-A*)	RSE	OC proportional odds (*BII-B*)	RSE	BI model without random effect in g() (*BII-C*)	RSE
Baseline	6.7	2.1%	-	-	0.0312	130.8%
Placebo effect	-1.5	19.2%	-1.44	15.8%	-0.316	19.4%
Drug effect	-0.577	56%	-0.595	43.2%	-0.141	44.5%
Standard deviation BI (g())	-	-	-	-	0.328	5.2%
IIV						
Baseline	228.3%	6%	213.3%	6.5%	43.8%	6.5%
Placebo effect	191.3%	11.6%	-	-	38.7%	11.5%
Baseline—placebo effect correlation	-32.5%	15.4%	-	-	-	-
Residual error						
Additive	147%	4.7%	-	-	-	-
OC parameters						
B1	-	-	5.98	4.4%	-	-
B2	-	-	-1.08	12.1%	-	-
B3	-	-	-1.06	11.4%	-	-
B4	-	-	-0.993	8.8%	-	-
B5	-	-	-0.968	8.7%	-	-
B6	-	-	-0.701	9.9%	-	-
B7	-	-	-0.704	9.1%	-	-
B8	-	-	-1.01	8.3%	-	-
B9	-	-	-1.54	8.6%	-	-
B10	-	-	-0.75	13.1%	-	-
B11	-	-	-1.03	14.3%	-	-
B12	-	-	-1.2	21.5%	-	-
B13	-	-	-2.79	26.1%	-	-

g() is the variance function in the bounded integer (BI) model. B_m_ is the baseline logit probability of a BII ≥ m (where m = 1,..,13). *IIV* inter-individual variability, *RSE* relative standard error

##### Bounded integer model

Including IIV for the g() parameter did not result in a statistically significant improvement in OFV, and therefore only a single BI model was developed for the BII scale. In the BII BI model (*BII-C*), incorporation of an offset drug effect decreased the OFV by 5.1. The parameter estimates for the BI model are shown in Table III. No dose-response or exposure-response model provided a significant decrease in OFV. A VPC for the BI BII model is shown in the Supplemental Material, indicating adequate fit of the model.

### Akaike information criterion

Table IV shows the AIC for all the developed models. Within the IPSS and QoL scales, BI models displayed a lower AIC compared to the reference models when a random effect was included in the BI variance function, g(). Furthermore, the BI model for the BII (that did not include a g() random effect) (*BII-C*) also showed a lower AIC compared to the CV BII model (*BII-A*), and so did OC QoL model (*QoL-A*) compared to the BI QoL model without a g() random effect (*QoL-B*). However, the CV IPSS model (*IPSS-A*) performed better in terms of fit compared to the BI model without a g() random effect (*IPSS-B*), and the same was seen with the OC BII model (*BII-B*) compared to the BI BII model (*BII-C*).

**Table IV:** Difference in Akaike information criterion (AIC) between reference and bounded integer (BI) models

Scale	AIC reference model(s)		AIC BI model without IIV in g()		∆AIC_Reference model_	AIC BI model with IIV in g()		∆AIC_Reference model_
IPSS	*IPSS-A*	16869.2	*IPSS-B*	17009.9	140.7	*IPSS-C*	16618.3	-250.9
						*IPSS-D*	16619.1	-250.1
QoL score	*QoL-A*	7702.1	*QoL-B*	7390.6	-311.5	*QoL-C*	6802.1	-900
BII	*BII-A*	5320.5	*BII-C*	5250.1	-70.7	-	-	-
	*BII-B*	5225.2	*BII-C*	5250.1	24.9	-	-	-

The reference models treated the International Prostate Symptom Score (IPSS) as a continuous variable (model *IPSS-A*) and the quality of life (QoL) score as ordered categorical (OC) (model *QoL-A*), while both a CV (model *BII-A*) and an OC model (model *BII-B*) were developed for the benign prostatic hyperplasia impact index (BII). AIC was calculated as the objective function value multiplied by two times the number of parameters. ∆AIC_reference_ was calculated as AIC_Bounded Integer Model_ – AIC_Reference Model_. g() is the variance function in the BI model. Two BI models with IIV in g() were developed for the IPSS scale: one with (model *IPSS-C*) and one without (model *IPSS-D*) the *Drift* parameter. *IIV* inter-individual variability

### Power and type I error in detecting a drug effect

**Fig. 1:**
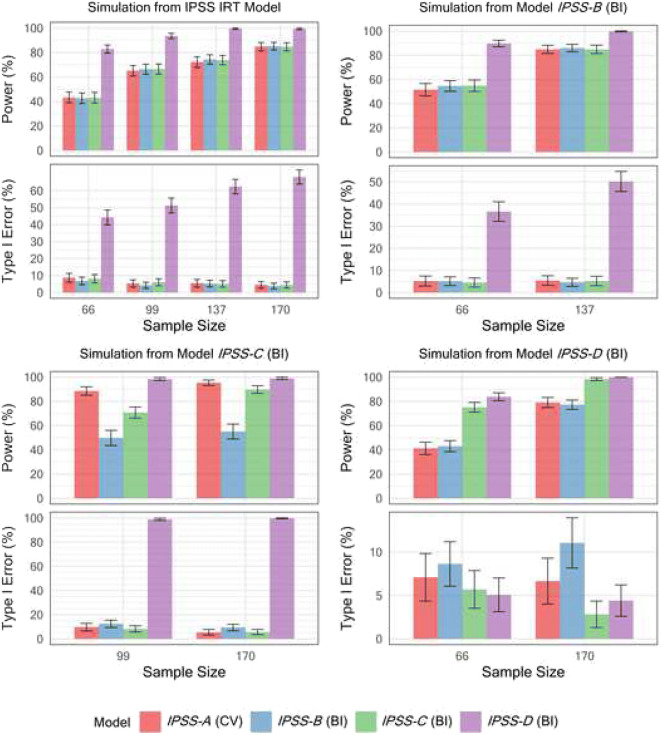
Power and type I error in detecting a drug effect for the four developed pharmacometric models describing the International Prostate Symptom Score (IPSS) under four different simulation models and varying trial sample sizes. Five hundred trial replicates were generated under each sample size for both power and type I error estimation. Model *IPSS-A* used a continuous variable (CV) approach with a combined residual error model. The bounded integer (BI) model *IPSS-B* did not contain inter-individual variability (IIV) in the BI variance function (g()), BI model *IPSS-C* contained both IIV in g() as well as a *Drift* parameter, while *IPSS-D* contained IIV in g() but did not estimate a *Drift* parameter.

**Fig. 2:**
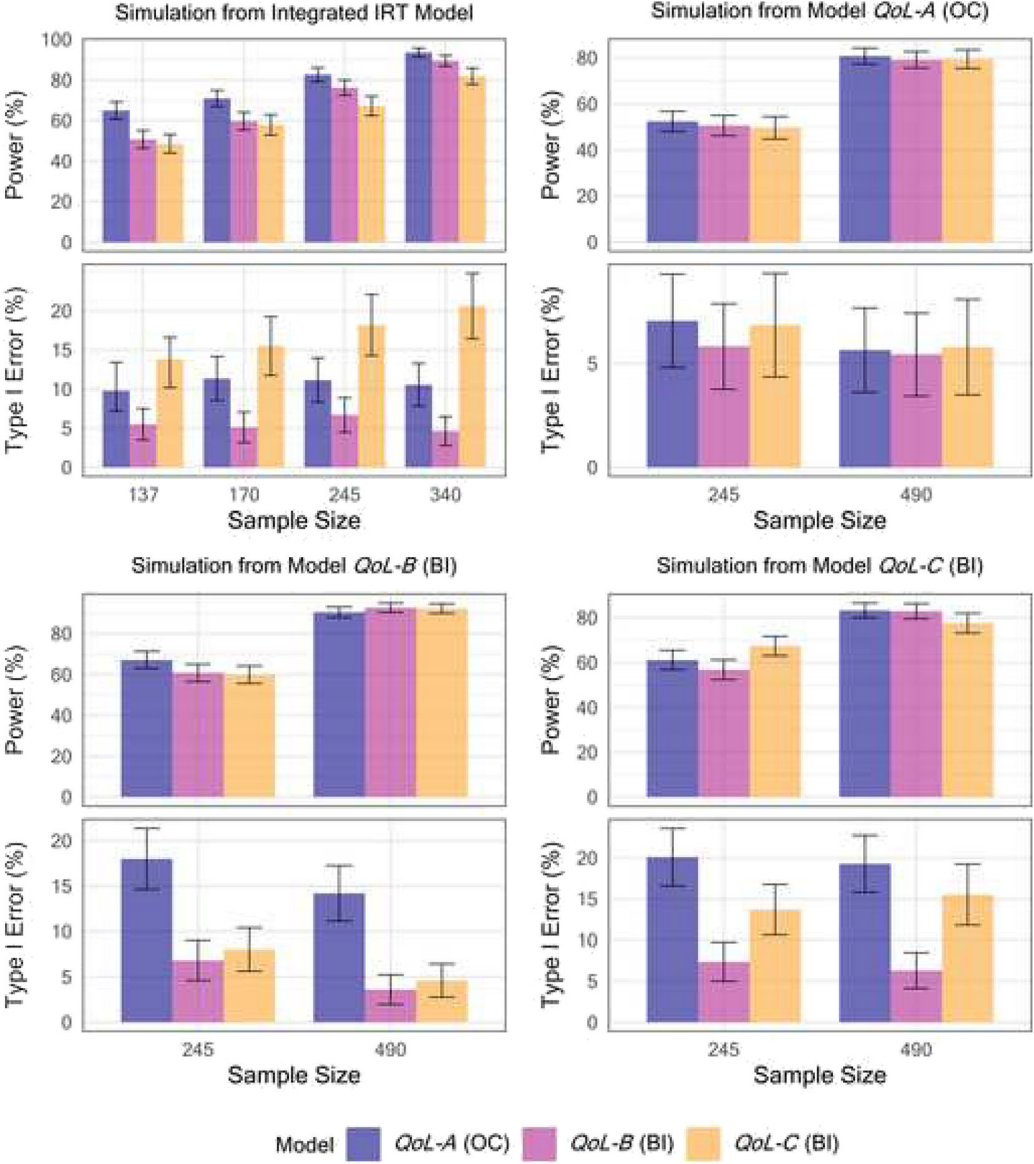
– Power and type I error in detecting a drug effect for the three developed pharmacometric models describing the Quality of Life (QoL) score under four different simulation models and varying trial sample sizes. 500 trial replicates were generated under each sample size for both power and type I error estimation. Model *QoL-A* used an ordered categorical (OC) approach. The bounded integer (BI) model *QoL-B* did not contain inter-individual variability in the BI variance function (g()) while BI model *QoL-C* did

The power to detect a drug effect and associated type I error of the four IPSS models under four different simulation scenarios is shown in Fig. 1. The type I error of detecting a drug effect of the BI model with IIV in g()and omitting the *Drift* parameter (model *IPSS-D*) was very high across all investigated simulation scenarios except for when data was simulated from model *IPSS-D* itself. On the other hand, the BI IPSS model that retained both the *Drift* parameter and the g() random effect (model *IPSS-C*) generally showed a type I error rate at the nominal level in all scenarios, similar to the CV IPSS model (*IPSS-A*). The BI IPSS model without a g() random effect (model *IPSS-B*) showed an adequate type I error control except when the data was simulated from BI models incorporating IIV in the g() function (i.e., simulation from models *IPSS-C* and *IPSS-D*, respectively, in Fig. 1). Overall, the CV model (model *IPSS-A*) showed the best type I error control and power across the different scenarios. Figure 2 shows the power and type I error of the developed QoL models. As reported in the model development section, these models did not include a drug effect parameter due to lack of statistical significance of the latter in the CS36 trial. A hypothetical offset drug effect parameter was therefore specified in the OC simulation model (*QoL-A*) as well as in the respective BI simulation models (*QoL-B* and *QoL-C*), similar to the offset drug effect included in the IRT simulation model (13). The magnitude of the drug effect was specified as -0.5 in OC simulation model *QoL-A* and -0.1 in the BI simulation model *QoL-B* and *QoL-C*, respectively. Consequently, an offset drug effect was also included and estimated in the full OC PO QoL score and the BI QoL score models in the SSE procedure in order to estimate type I error and power. BI model *QoL-B* showed the best type I error control among the three models across all four simulation scenarios, as the 95% CI for the type I error estimate consistently included 5%. Meanwhile, the OC model *QoL-A* and the BI model with a random effect in g() *QoL-C* had highly inflated type I error in detecting a drug effect, except when data was simulated from the OC model *QoL-A*.

**Fig. 3:**
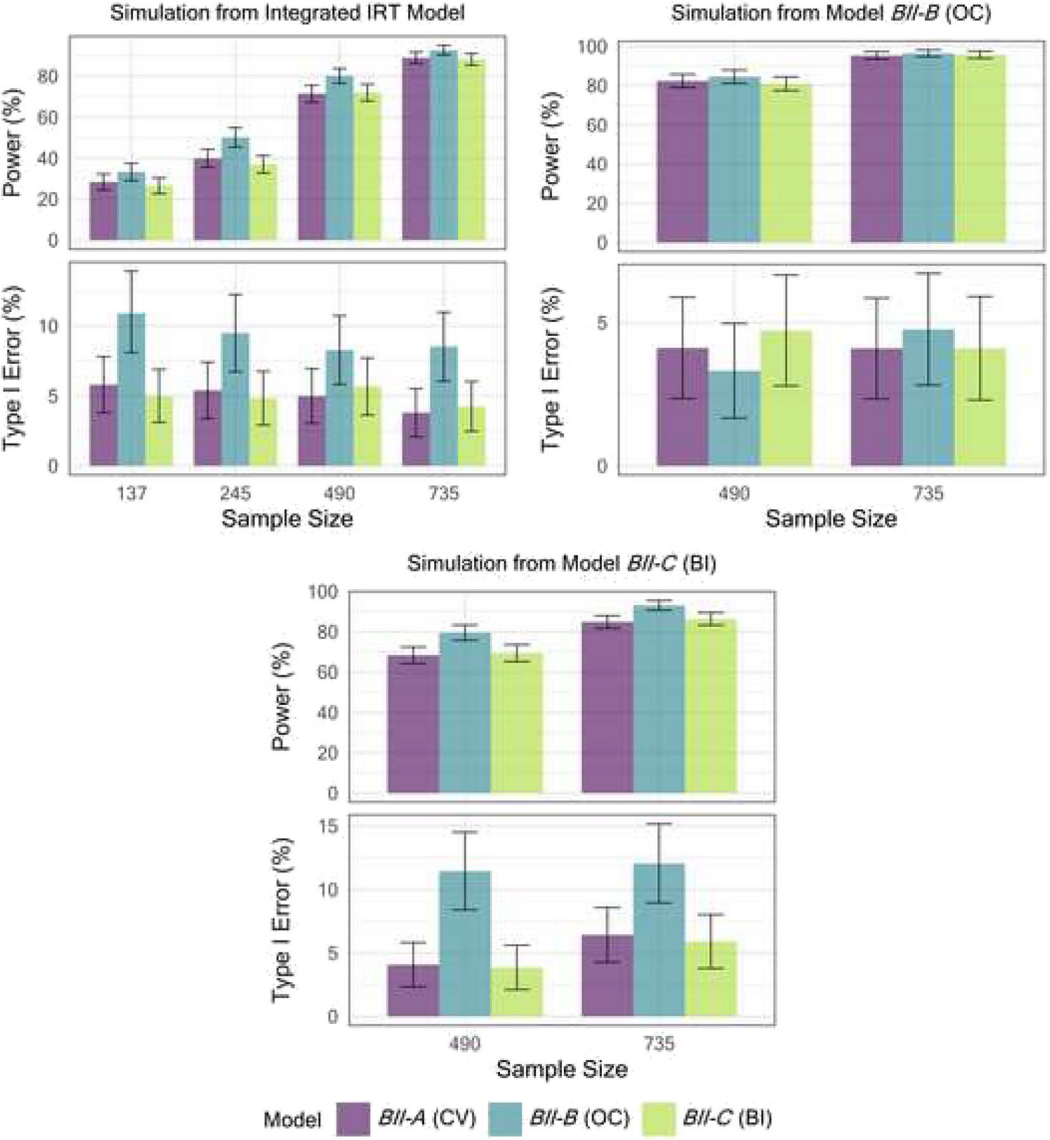
Power and type I error in detecting a drug effect for the three developed pharmacometric models describing the BPH impact index (BII) score under three different simulation models and varying trial sample sizes. Five hundred trial replicates were generated under each sample size for both power and type I error estimation. Model *BII-A* used a continuous variable (CV) approach with an additive residual error model. Model *BII-B* used an ordered categorical (OC) approach. Bounded Integer (BI) model *BII-C* did not contain inter-individual variability in the BI variance function (g())

Figure 3 shows the power and type I error of models for the BII scale under three different simulation scenarios. The type I error and power of the CV BII model (*BII-A*) and BI model *BII-C* that did not contain IIV in g() was comparable in all simulated scenarios, while the OC BII model (BII-B) showed inflated type I error except when data was simulated from this model (Fig. 3, top right panel).

### Joint bounded integer modeling

**Fig. 4:**
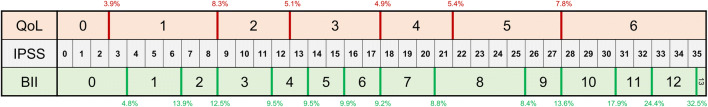
Schematic representation of the relationship between scores of the International Prostate Symptom Score (IPSS), quality of life (QoL), and the benign prostatic hyperplasia impact index (BII) scales in the joint bounded integer model. The probits of the IPSS were used as reference cut-offs. The bold red and green vertical lines indicate the estimated cut-offs for the QoL and BII scores, respectively, along with their relative standard errors. Back translation of latent *z*-score values to observe score values allowed for mapping of scores

A joint BI model describing responses to the IPSS, QoL score, and BII over time in 403 patients was successfully developed. A longitudinal model similar to the BI IPSS model with a random effect in g() and without a drift parameter (*IPSS-D*) described the data. Incorporation of a separate BI variance function for the BII scale decreased the objective function by 583.5 points. An offset drug effect was estimated yielding an OFV drop of 50.5 points. The relationship between scores on each scale was estimated with overall low uncertainty as shown in Fig. 4. The estimated cut-offs allowing translation between the IPSS and the QoL score all had low uncertainty associated with them (< 8% RSE). Higher uncertainty (< 14% RSE) was observed for the cut-offs relating the IPSS to the BII compared to the IPSS-QoL cut-offs, and this was especially pronounced for BII scores greater than 10 (RSE ranging from 17.9 to 32.5%). The longitudinal parameter estimates of the joint BI model are shown in the Supplemental Material. All three scales were adequately described in the joint BI model as assessed through VPCs. These are shown in the Supplemental Material along with the NONMEM code used to generate the joint BI model.

## Discussion

This study presents the application of BI modeling within a BPH-LUTS clinical trial setting, where multiple disease-specific scale endpoints traditionally assess the efficacy of new treatments. Building further upon previous investigations focusing on AIC comparison of BI models with CV and OC models, respectively (11), the current study assesses the power and type I error in detecting a drug effect of the BI modeling approach compared to these traditional methods. Lastly, by way of development of a joint BI model, the relationship between scores of the IPSS, QoL, and BII scales was established, which to our knowledge has not been shown beforehand.

### Akaike information criterion

The first objective of the current paper was to develop BI models for each of the three BPH-LUTS scales used in trial CS36. Models with and without IIV in g() were developed, and this led to the development of three different BI models for the IPSS (*IPSS-B*, *IPSS-C*, and *IPSS-D*), two for the QoL score (*QoL-B* and *QoL-C*), and one for the BII scale (*BII-C*). Similar to previous BI models of different scales (11), the BI models with IIV in g() (models *IPSS-C*, *IPSS-D*, and *QoL-C*) showed the best data description in terms of AIC among all models within each scale. It can however be argued that since including IIV in the BI variance function translates to incorporating IIV in the residual variance of a CV model (11), this would be a fairer comparison. Hence, the CV IPSS model (model *IPSS-A*) was exploratorily further developed to include IIV in the residual variance (which we in the “Discussion” section refer to as model *IPSS-A-2*). An additive error model and a log-normally distributed IIV similar to the implementation by Karlsson *et al.* (17) were used, as the combined error model resulted in convergence issues. Furthermore, similar to model *IPSS-D*, the *Drift* parameter was no longer statistically significant and was removed from the model. Model *IPSS-A-2* showed a substantially lower AIC compared to the BI IPSS model with a g() random effect (decrease of 789.2 points). However, its simulation properties were poor, as substantial data was simulated well below and above the possible minimally and maximally possible IPSS of zero and 35, respectively (Supplemental Material). Hence, compared to the CV approach which implements an additional random effect in the residual error, BI modeling with IIV in g() may have the advantage of describing the data well while preserving high predictive ability.

Unlike the previous analysis of the Likert pain data (11), the BI model of the QoL score without an IIV random effect in g() (model *QoL-B*) showed substantially better fit in terms of AIC compared to the OC model (model *QoL-A*). However, the fit of the BI BII model without IIV in g() (model *BII-C*) was not superior to the OC BII model (*BII-B*). The current data had eight measurements for the QoL score and three BII measurements per patient over the 6-month trial period. The number of longitudinal measurements may hence potentially influence the descriptive performance of BI modeling compared to the OC method; as the number of visits increases, more complex longitudinal trajectories may be captured by the BI model. The latter allows for inclusion of more IIV components compared to the standard OC model with a single random effect. Further discussion on comparison of BI and OC modeling has been reported elsewhere (11).

In previous work, BI models were reported to result in certain parameters becoming “superfluous” compared to their corresponding CV models (11). In the current work, however, the same structural model was ultimately developed for the BI model without IIV in g() (model *IPSS-B*) and the CV IPSS model (*IPSS-A*). However, following introduction of IIV in g() during BI IPSS modeling (models *IPSS-C* and *IPSS-D*), the *Drift* parameter was no longer statistically significant. The introduction of an IIV random effect in g() may therefore be a source of explanation for parameters losing statistical significance, as its flexibility may affect parameter identifiability.

### Type I error and power

The second objective of the current analysis was to investigate the type I error and power of BI models, and this was achieved through a series of different simulations within each scale. Results overall indicated that incorporating IIV in the g() function of BI models may result in very high type I error as evidenced within different simulation scenarios for the IPSS and for the QoL scale. In the IPSS simulations, the increase in type I error may be attributed to model misspecification: When simulating from models that incorporate the *Drift* parameter (the IPSS IRT model, model *IPSS-B*, and model *IPSS-C* in Fig. 1), the BI model without this parameter (*IPSS-D*) consistently showed severely inflated type I error. When simulating from model *IPSS-D*, this same model performed well in terms of type I error and showed higher power to detect a drug effect compared to other models (*IPSS-A*, *IPSS-B*, *IPSS-C*); however, it is to be noted that the latter models still controlled type I error adequately (except for model *IPSS-B*, which had slightly inflated type I error). In the case of QoL score simulations, the BI model with a g() random effect (model *QoL-C*) had high η-shrinkage (> 80%) in the *Tprog* IIV term. This likely rendered this IIV term largely uninformative due to model parameter identifiability issues, thereby resulting in model misspecification. Furthermore, for models *IPSS-D* and *QoL-C* (with IIV in g()), the type I error was seen to increase with increasing sample size, namely, in the IRT simulation scenarios in Figs. 1 and 2, respectively. This may indicate that the more patients are included in the trial (and hence the larger the IIV in the longitudinal trajectories of patients), the larger room for error in detecting a drug effect with this type of BI model. It is however to be noted that the type I error of an exploratory CV model with IIV in the residual variance (model *IPSS-A-2* presented earlier in the “Discussion” section) was similar to model *IPSS-D* across the four different simulation scenarios (data not shown). The severely inflated type I error as observed with model *IPSS-D* may therefore not be exclusive to the BI approach, but may instead be associated with incorporating IIV in the residual error component of models. Including IIV in the residual variance has shown adequate type I error control in terms of covariate inclusion in PK models (18). However, to our knowledge, it has not previously been investigated within the context of detecting drug effects in longitudinal drug-disease modeling using a nonlinear mixed effects modeling framework. Further research on these types of models may be of interest.

Misspecification of random effects is known to increase type I error for hypothesis testing on the fixed effect of interest within the context of linear mixed models (19,20). Authors have furthermore pointed towards data-driven random effects specification having higher potential for type I error inflation compared to the design-driven approach (20–22). This may also apply to the current findings; e.g., in the IRT simulation scenarios, the CV (*IPSS-A*) and BI models (*IPSS-B*, *IPSS-C*, and *QoL-B*) included similar random effects as the simulation models (12,13). The developed BI models were therefore more in line with the design of the simulated trial data and consequently in general showed better type I error rates. Oppositely, the IPSS BI model that included IIV in g() and omitted the *Drift* IIV parameter (model *IPSS-D*) could be said to adhere to a data-driven random effect specification approach. The IRT models used for simulation (12,13) were previously developed on the same CS36 data set as the current models, explaining the similarity between random effects.

To preserve both good data description as well as adequate type I error control in detecting a drug effect, it could be advised that if IIV is to be included in the BI g() function, this should be performed as the last model development step. Careful consideration of the influence of this parameter on the original structural model parameters (i.e., without IIV in g()) should be given, e.g., η-shrinkage. Furthermore, parameters that were significant prior to inclusion of IIV in g() may have to remain in the model regardless of their significance after its inclusion (e.g., similar to the *Drift* parameter in model *IPSS-C*). However, although this proposed approach may afford better type I error control, it may also limit the power to detect a drug effect: This was observed with model *IPSS-C* when simulating data from models *IPSS-C* and *IPSS-D*, respectively, in Fig. 1. This loss of power was also seen with models *IPSS-A*, *IPSS-B*, and *IPSS-C* when simulating from model *IPSS-D* with no *Drift* parameter.

Even though the IPSS CV model (*IPSS-A*) inherently violates the integer nature of the scale data, it was overall more robust in terms of type I error control and preservation of power compared to the different BI models across the different IPSS simulation scenarios. Notably and surprisingly, when simulating from BI model *IPSS-C*, the CV model (*IPSS-A*) had substantially higher power compared to BI model *IPSS-B* although they both shared a similar longitudinal model structure. Furthermore, model *IPSS-A* had higher power to detect a drug effect than the BI simulation model *IPSS-C*. A potential explanation may be that probabilistic models such as BI (and OC) do not incorporate residual error in the same manner as the CV approach. The residual error component in CV models may be an important factor in terms of signal-to-noise, as it describes residual error through a random effect (or two in the case of a combined error model) and thereby differs from the standard BI fixed effect g() function (e.g., as in model *IPSS-B*). Model *IPSS-A* implemented a combined residual error model, and in sensitivity analyses, similar power and type I error was observed with a CV model implementing an additive residual error model (data not shown). CV modeling of bounded scores using a combined error model has previously been reported to result in ill behavior (23). However, this was not the case in the current work, as model *IPSS-A* converged successfully, yielded a lower AIC than implementing an additive residual error model, and did not suffer in terms of type I error and power performance in detecting a drug effect. Lastly, the loss of power in *IPSS-C* may potentially be explained by the very high correlation between random effect parameters *Drift* and *Asy* in model *IPSS-C*, hindering clear distinction between these parameters during estimation, and thereby limiting the power to detect a drug effect.

The BI QoL model with no random effect in g() (model *QoL-B*) showed the best type I error control with no loss of power across the different QoL score simulation scenarios (Fig. 2), highlighting that this type of BI approach may be well-suited for detecting drug effects in trials using smaller scales for which using a CV approach is not viable. The poor type I error control observed with the OC models of the QoL score (*QoL-A*) and the BII (*BII-B*), respectively, may be explained by the limited number of IIV parameters that may be incorporated into OC models. A standard OC PO model with a single random effect on the baseline logit probability was used in the current work. BI models may allow for a greater number of IIV parameters to be incorporated and hence potentially would allow for more flexibility for describing heterogeneous and stochastic data while accurately detecting drug effects.

Meaningful comparison of the power to detect a drug effect between models can only be made when their type I error rate is comparable. Therefore, no comparison of power was made when the type I error of models differed substantially. Adjustment for type I error was performed for power comparison in an exploratory fashion (data not shown), yet accurate determination of type I error adjusted power may require many more trial replicates (e.g., ~ 10,000) (24).

This work is the first to investigate the power and type I error of the BI approach. The overall findings indicate that the methodology performs well in comparison to traditional methods when no IIV is included in the g() function. However, further research to optimize its performance in detecting drug effects may be required, particularly when including IIV in the g() variance function. Individual model averaging (25,26), which has recently shown high ability to control the type I error of detecting a drug effect in the presence of model misspecification, may be of interest to investigate in this context. Moreover, in the original presentation of the BI methodology (11) as well as in the current study, probits as driven by the standard normal distribution determined the score cut-offs, and a normal distribution described the mean-variance (f()-g()) function. The impact on type I error and power when using other distribution functions in BI models as well as further developments to the BI g() function may be of interest to inspect. Moreover, as other methods for analyzing bounded score data have been presented, such as beta-regression (27), censoring (28), and the coarsened grid (29), the current results emphasize that further research examining the power and type I error of these methods is also of interest. Lastly, the current simulation scenarios assumed a parallel-group placebo-controlled trial design spanning a 6-month period, similar to the CS36 trial. Offset drug effects were also exclusively investigated in the simulation scenarios. Future research may potentially seek to investigate the performance of models while varying trial design characteristics (such as study duration) and other types of drug effects.

### Joint bounded integer model

A joint BPH-LUTS scale BI model was developed, incorporating responses to the IPSS, QoL score, and BII in individual patients over the 6-month trial period. This allowed for quantification of the connection between scores on each scale and thus achieved the third objective of the current paper. Knowledge of the relationship between scores on different BPH-LUTS scales may be useful, e.g., for comparison of patient population characteristics in different BPH-LUTS clinical trials, where trial inclusion is commonly contingent on patients’ response to one or more BPH-LUTS scales and may differ substantially between trials. In clinical diagnosis of BPH-LUTS, three categories of BPH-LUTS severity have been specified based on the IPSS: mild (0 to 7), moderate (8 to 19), and severe (20 to 35) (3). In the current model, it was estimated that mild BPH-LUTS translates to a QoL score ≤ 1 or a BII ≤ 2, moderate BPH-LUTS translates to a QoL score of 2 or 3 or a BII of at least 3 and maximally 6, and lastly, severe BPH-LUTS translates to a QoL score ≥ 4 or a BII ≥ 7. Furthermore, it was estimated that an IPSS ≥ 13 corresponds to a QoL score ≥ 3, which is consistent with the inclusion criteria used in the currently analyzed trial, as well as many other BPH-LUTS clinical trials. Within the scope of BPH-LUTS clinical trials designed in similar fashion to the CS36 trial, the joint BI model may also be used for predictive purposes. For example, it may be served in obtaining knowledge regarding the longitudinal trajectory of the QoL and BII in patients should only IPSS data be available from patients. Given that similar longitudinal parameter estimates were obtained in the IPSS BI approach (model *IPSS-D*) and in the joint BI model, prediction of QoL and BII data may be achieved through initial development of an IPSS BI model followed by input of the longitudinal parameter estimates into a joint BI model to be used for simulation. The currently reported cut-off estimates for the QoL score and the BII (as well as the latter scale’s separate variance g() function) could then be used to simulate these scores. It is to be noted that using a joint BI modeling approach may serve as the primary modeling strategy (instead of developing separate BI models for each scale) and has been utilized in previous work (15,30). Use of a joint BI approach is also supported in the current work given the adequate description of data from each scale as assessed through VPCs. Ultimately, the choice between individual and joint BI modeling likely depends on the goal of the pharmacometric analysis, and further research may seek to emphasize the benefits of each of the two approaches depending on the latter.

The relationship between the IPSS and QoL scores in the joint BI model was estimated with low uncertainty, while larger imprecision was observed for the estimated BII cut-offs. This may be explained by the fewer observed BII measurements per patient compared to the QoL in the current data (three versus eight measurements per patient over the 6-month trial period). The high uncertainty was especially evident for cut-offs for BII scores above 10, which may be explained by fewer of these scores being observed in the current data set. Another way to connect scores on different scales is by the use of IRT modeling, where patients’ underlying disability serves as the link between scales. Good alignment with the results of such analysis (13) was seen with the current BI approach. Lastly, covariate relationships may be of interest to investigate to further improve the joint BI model. Due to long run times with the current model, this was not performed in this study.

## Conclusion

This paper presents the development of BI models for three different commonly used scales within BPH-LUTS based on data from a Phase II clinical trial. Through simulations, this study sheds light on the type I error and power to detect a drug effect of BI modeling in comparison to traditional methods for analyzing bounded score data from different BPH-LUTS scales. Overall, the CV approach was more robust compared to the BI approach although it violates the integer nature of the data. BI modeling without IIV in the variance function performed similarly to the CV approach in most cases; however, further research may seek to optimize its performance. Further research on type I error control of BI models with IIV in the BI variance function may be of high interest. In general, the OC approach showed higher type I error in detecting a drug effect compared to the BI approach, and the latter may therefore be an attractive approach for detecting drug effects in longitudinal analysis of trials using scales with few categories as endpoints. Lastly, a joint BI model was allowed for estimation of the relationship between scores of the IPSS, QoL, and BII scales, which may be useful knowledge in clinical diagnosis and translation between clinical trial inclusion criteria and results.

## Supplementary Information

ESM 1 (PDF 2012 kb)
