# Supplementary material for: Bounded Integer Modeling of Symptom Scales Specific to Lower Urinary Tract Symptoms Secondary to Benign Prostatic Hyperplasia
Source: AAPS J. 2021 Feb 25;23(2):33. doi: 10.1208/s12248-021-00568-y (PMC7906927; doi:10.1208/s12248-021-00568-y)
Supplement: Supplementary file 1 — (PDF 2012 kb) [file 12248_2021_568_MOESM1_ESM.pdf]

Supplemental Material

Bounded Integer Modeling of Symptom Scales specific to Lower Urinary Tract Symptoms Secondary to Benign Prostatic Hyperplasia

Contents

1. Visual predictive checks ... 2
   1.1 Bounded Integer models of individual scales ... 2
      1.1.1 International Prostate Symptom Score model without random effect in g() (*IPSS-B*) ... 2
      1.1.2 International Prostate Symptom Score model with random effect in g() and Drift (*IPSS-C*) ... 3
      1.1.3 International Prostate Symptom Score model with random effect in g() (*IPSS-D*) ... 4
      1.1.4 Quality of Life score model without random effect in g() (*QoL-B*) ... 5
      1.1.5 Quality of Life score model with random effect in g() (*QoL-C*) ... 6
      1.1.6 Benign Prostatic Hyperplasia Impact Index (*BII-C*) ... 7
   1.2 Continuous variable models ... 8
      1.2.1 International Prostate Symptom Score (*IPSS-A)* ... 8
      1.2.2 Benign Prostatic Hyperplasia Impact Index (*BII-A)* ... 9
   1.3 Ordered categorical models ... 10
      1.3.1 Quality of Life score (*QoL-A*) ... 10
      1.3.2. Benign Prostatic Hyperplasia Impact Index (*BII-B*) ... 11
   1.4 Joint Bounded Integer Model ... 12
      1.4.1 International Prostate Symptom Score ... 12
      1.4.2 Quality of Life score ... 13
      1.4.3 Benign Prostatic Hyperplasia Impact Index ... 14
   1.5 Exploratory Models ... 15
      1.5.1 IPSS Continuous variable with random effect in residual variance ... 15
2. Model parameter estimates ... 16
   2.1 Joint Bounded Integer Model ... 16
3. Model code ... 17
   3.1 Bounded Integer Model for the International prostate Symptom Score (*IPSS-C*) ... 17
   3.2 Joint Bounded Integer Model ... 23

1. Visual predictive checks

1.1 Bounded Integer models of individual scales

1.1.1 International Prostate Symptom Score model without random effect in g() (*IPSS-B*)

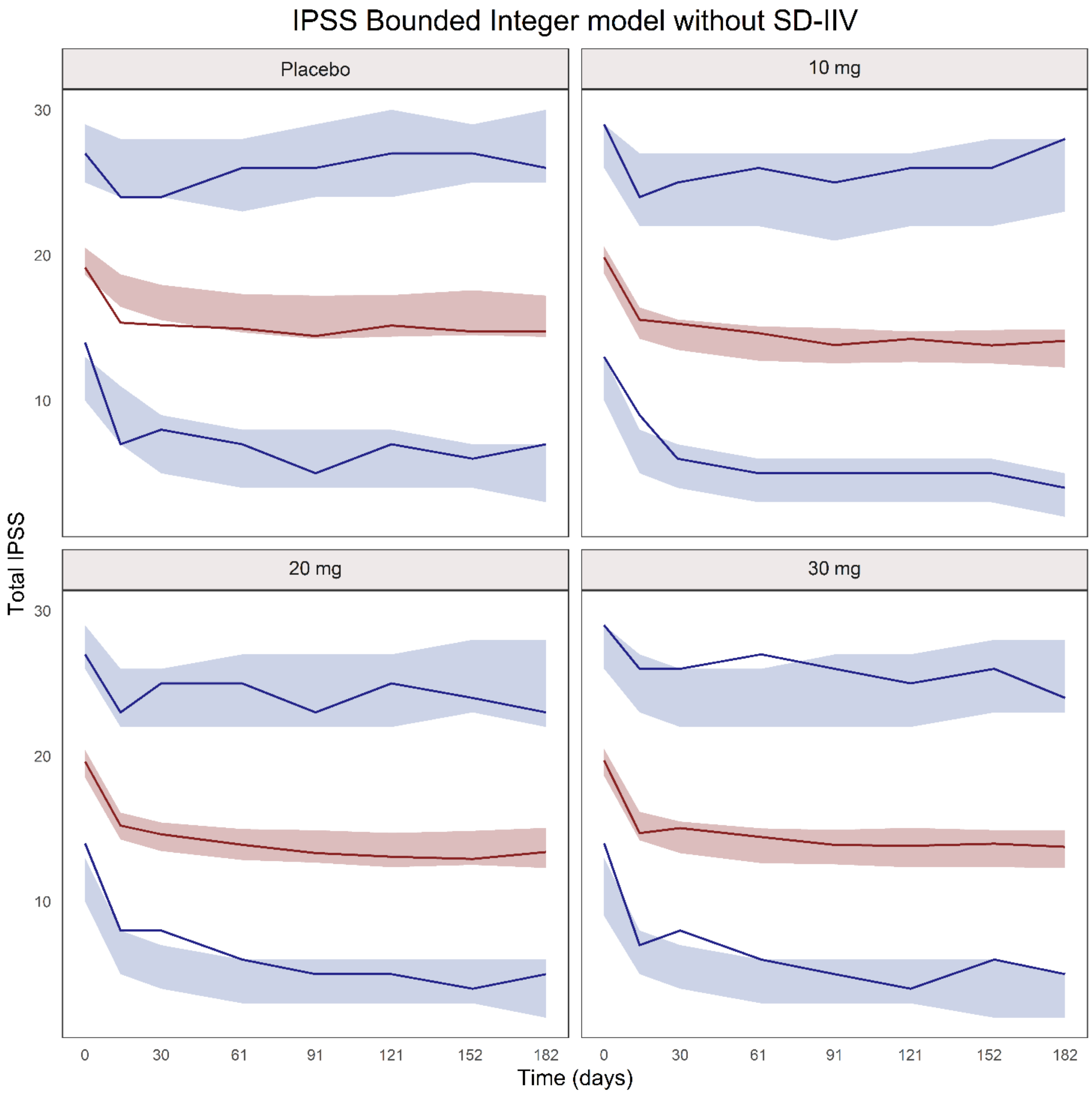

Figure S1.1.1 - Visual predictive check for the International Prostate Symptom Score (IPSS) bounded integer model without interindividual variability (IIV) in g() (SD) stratified by treatment arm comparing the median, 2.5th, and 97.5th percentiles of the observed data with the corresponding percentiles for simulated data displayed as 95% confidence intervals. Treatment effect was modeled as absent (placebo arm) or present (10 mg, 20 mg, and 30 mg degarelix arms). Reference model name: *IPSS-B*.

1.1.2 International Prostate Symptom Score model with random effect in g() and Drift (*IPSS-C*)

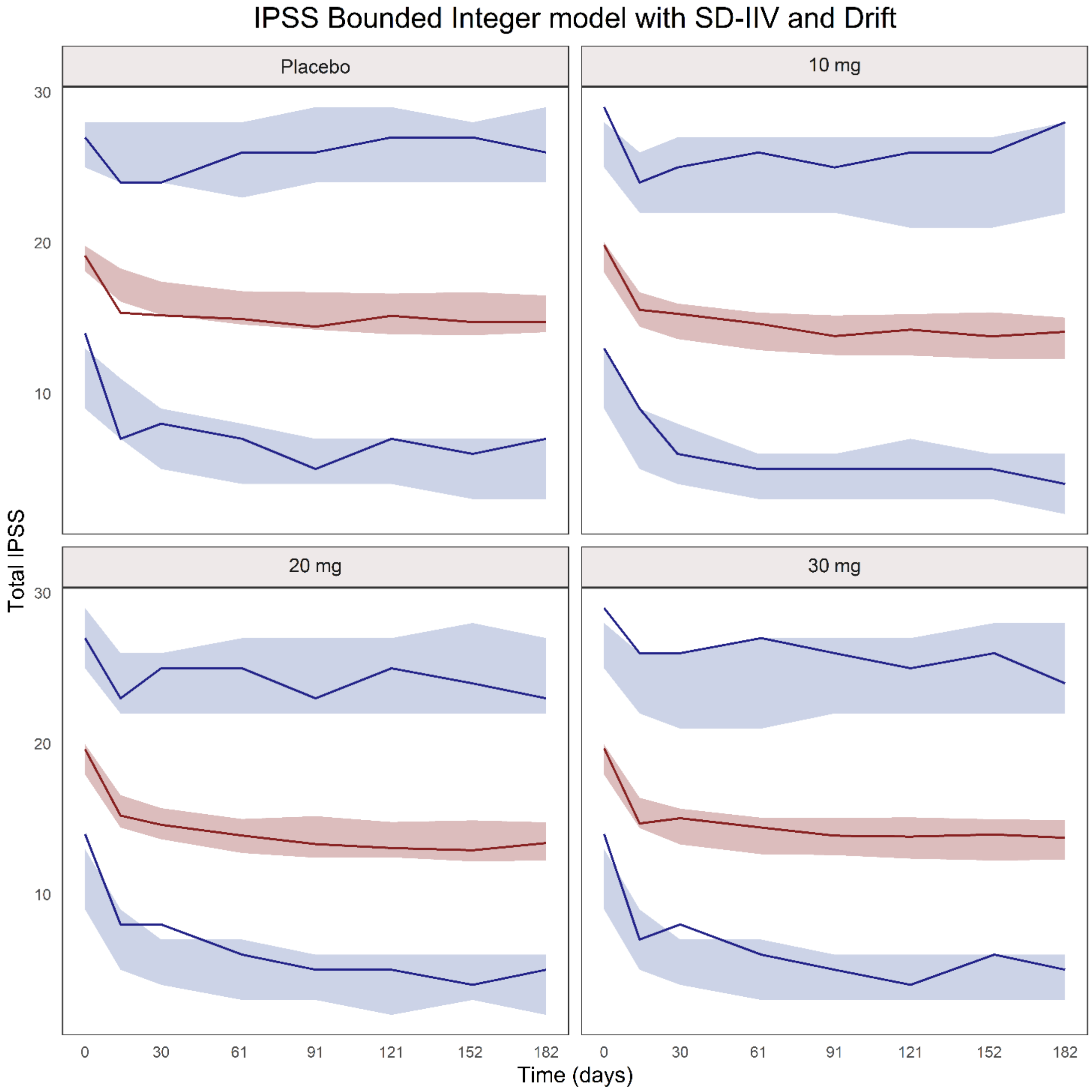

Figure S1.1.2 - Visual predictive check for the International Prostate Symptom Score (IPSS) bounded integer model with interindividual variability (IIV) in g() (SD) and a *Drift* parameter stratified by treatment arm comparing the median, 2.5th, and 97.5th percentiles of the observed data with the corresponding percentiles for simulated data displayed as 95% confidence intervals. Treatment effect was modeled as absent (placebo arm) or present (10 mg, 20 mg, and 30 mg degarelix arms). Reference model name: *IPSS-C*.

1.1.3 International Prostate Symptom Score model with random effect in g() (*IPSS-D*)

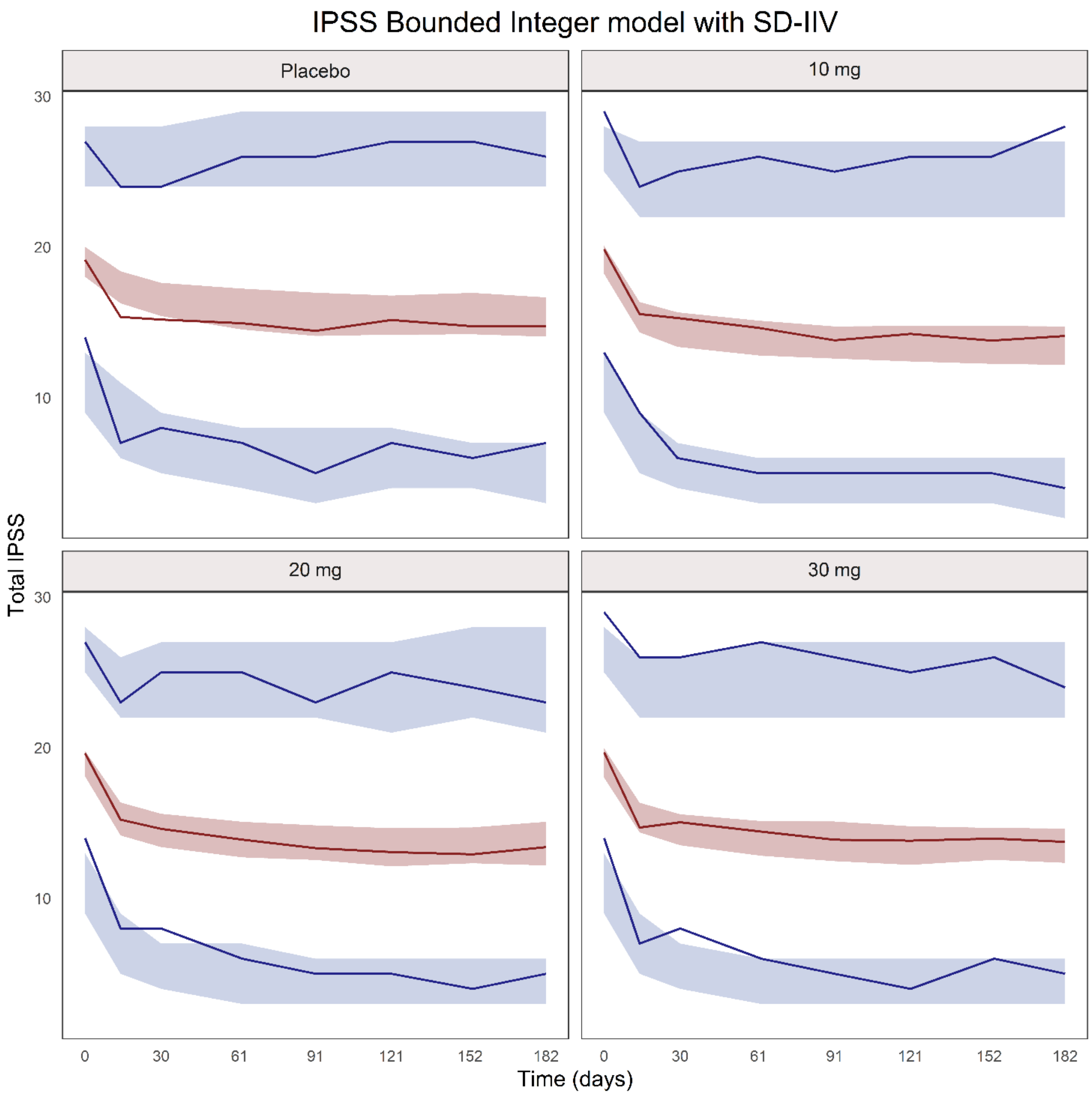

Figure S1.1.3 - Visual predictive check for the bounded integer model with the International Prostate Symptom Score (IPSS) interindividual variability (IIV) in g() (SD) stratified by treatment arm comparing the median, 2.5th, and 97.5th percentiles of the observed data with the corresponding percentiles for simulated data displayed as 95% confidence intervals. Treatment effect was modeled as absent (placebo arm) or present (10 mg, 20 mg, and 30 mg degarelix arms). Reference model name: *IPSS-D*.

1.1.4 Quality of Life score model without random effect in g() (*QoL-B*)

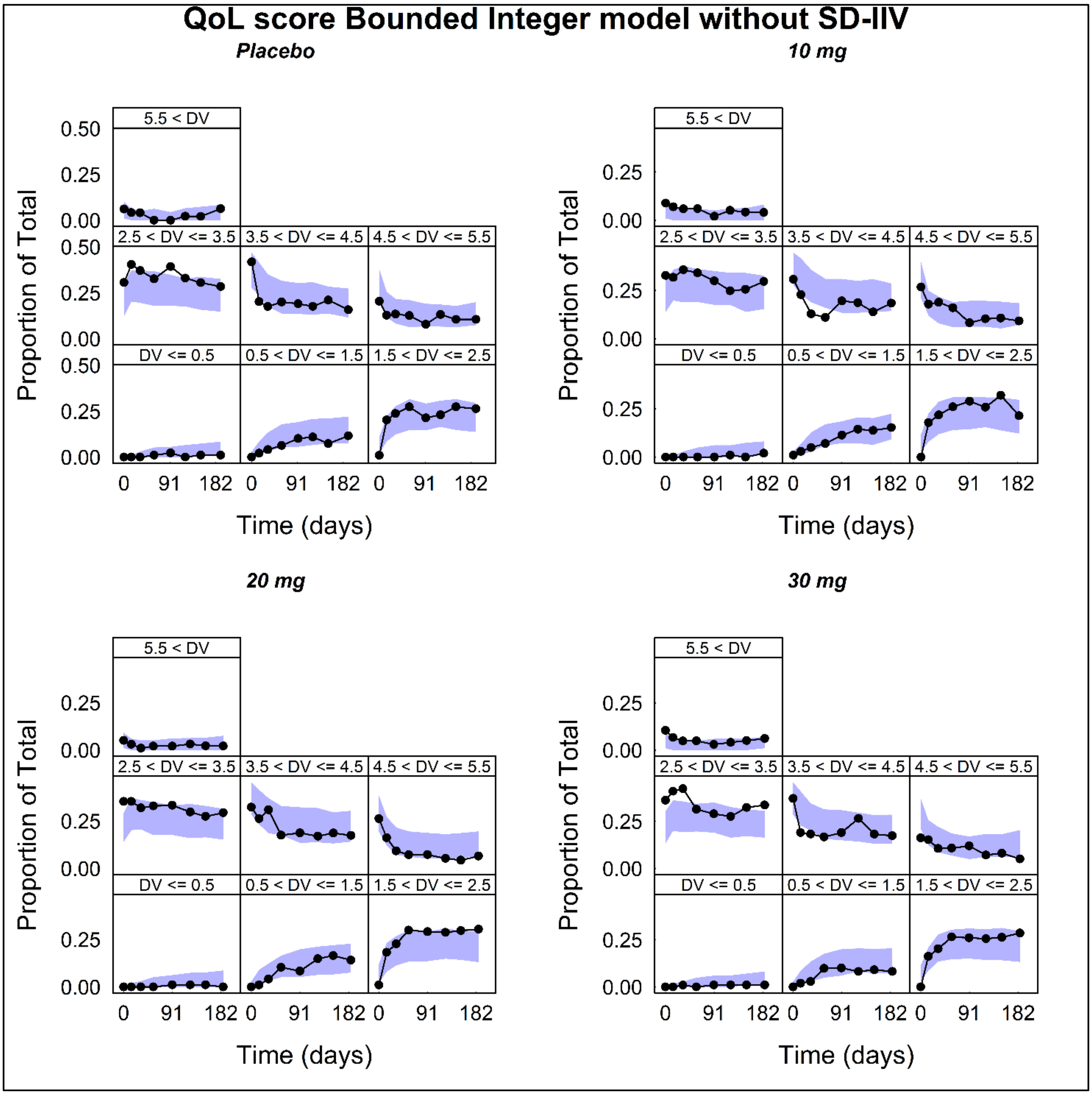

Figure S1.1.4 –Visual predictive check for the bounded integer Quality of Life (QoL) score model without inter-individual variability (IIV) in the g() function (SD) stratified by treatment arm. The observed frequency of each score over time is shown as points and the shaded areas indicate the 95% confidence intervals of the frequencies of each score in 200 simulated datasets. DV: Dependent variable, i.e. observed score.

1.1.5 Quality of Life score model with random effect in g() (*QoL-C*)

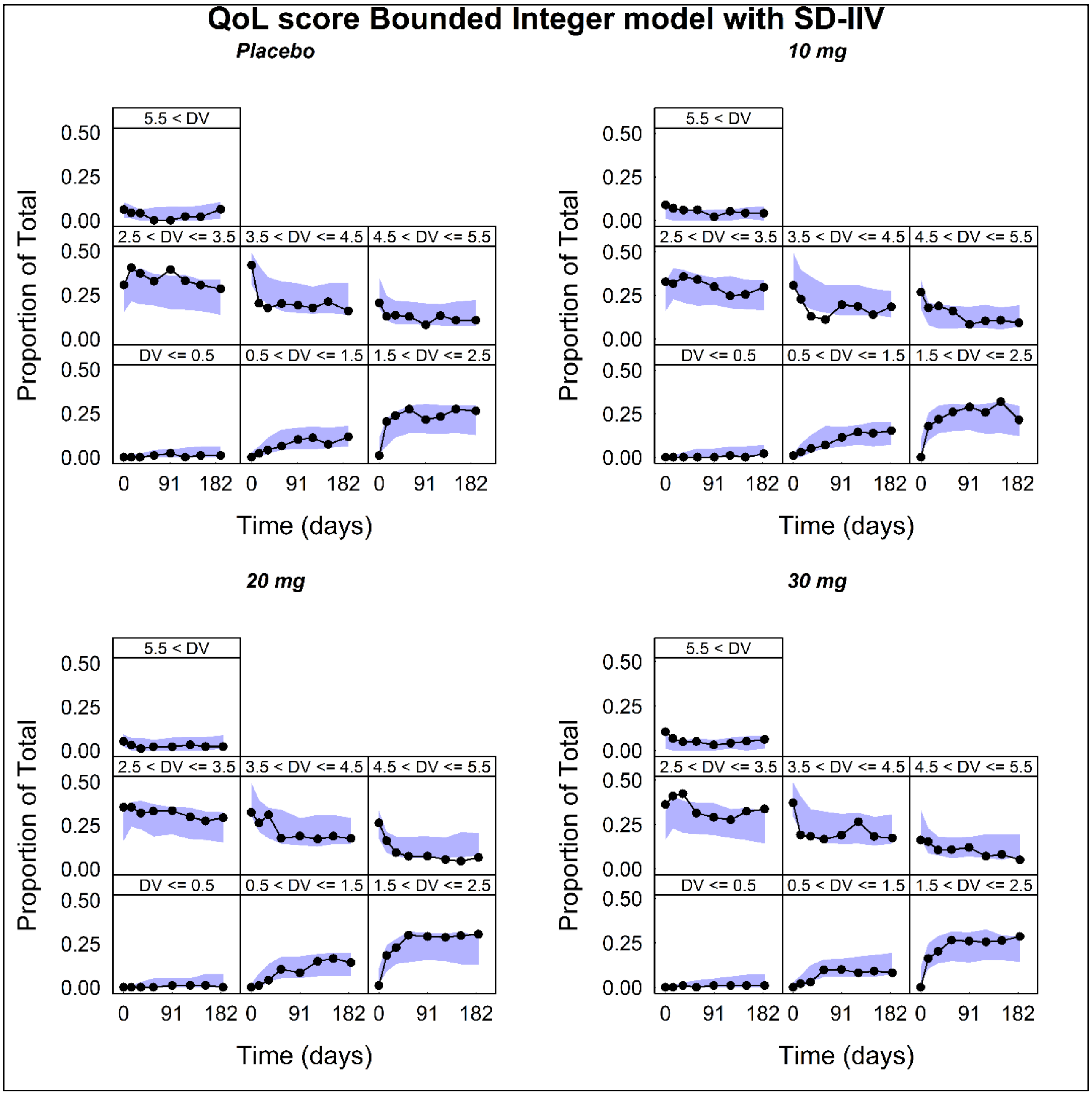

Figure S1.1.5 –Visual predictive check for the bounded integer Quality of Life (QoL) score model with inter-individual variability (IIV) in the g() function (SD) stratified by treatment arm. The observed frequency of each score over time is shown as points and the shaded areas indicate the 95% confidence intervals of the frequencies of each score in 200 simulated datasets. DV: Dependent variable, i.e. observed score. Reference model name: *QoL-C*.

1.1.6 Benign Prostatic Hyperplasia Impact Index (*BII-C*)

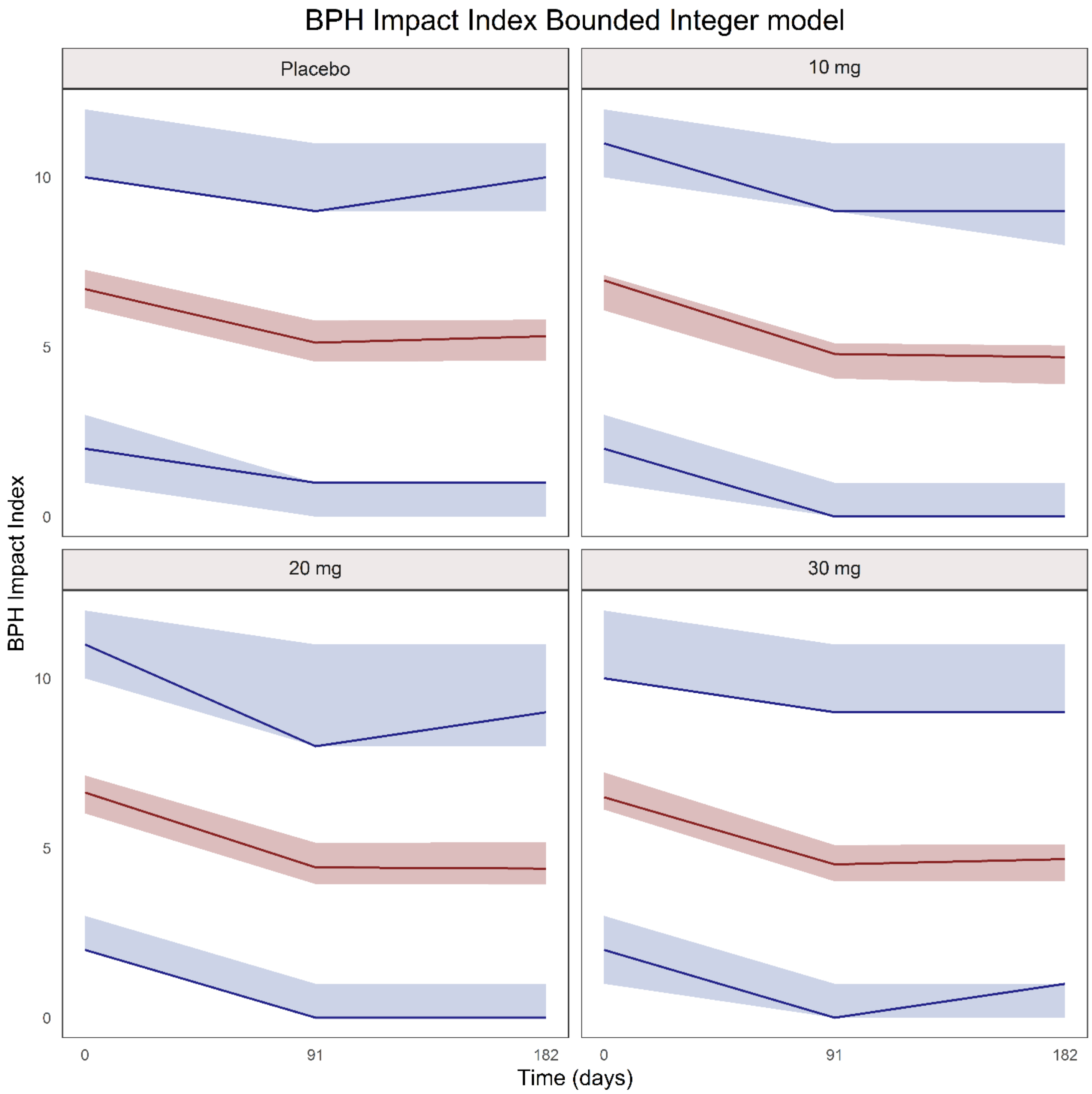

Figure S1.1.6 - Visual predictive check for the benign prostatic hyperplasia (BPH) impact index bounded integer model stratified by treatment arm comparing the median, 2.5th, and 97.5th percentiles of the observed data with the corresponding percentiles for simulated data displayed as 95% confidence intervals. Treatment effect was modeled as absent (placebo arm) or present (10 mg, 20 mg, and 30 mg degarelix arms). Reference model name: *BII-C*.

1.2 Continuous variable models

1.2.1 International Prostate Symptom Score (*IPSS-A)*

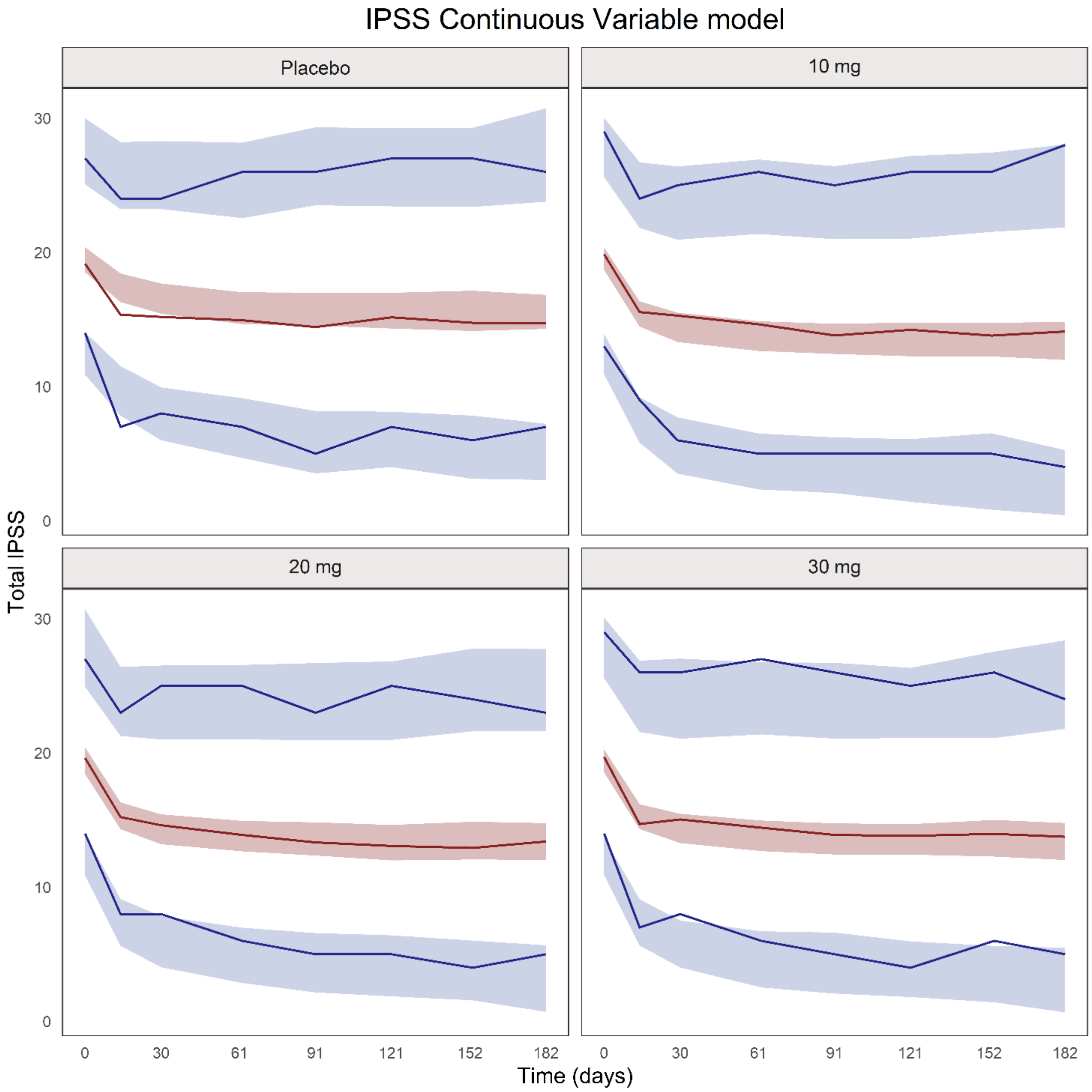

Figure S1.2.1 - Visual predictive check for the International Prostate Symptom Score (IPSS) continuous variable model stratified by treatment arm comparing the median, 2.5th, and 97.5th percentiles of the observed data with the corresponding percentiles for simulated data displayed as 95% confidence intervals. Treatment effect was modeled as absent (placebo arm) or present (10 mg, 20 mg, and 30 mg degarelix arms). Reference model name: *IPSS-A*.

1.2.2 Benign Prostatic Hyperplasia Impact Index (*BII-A)*

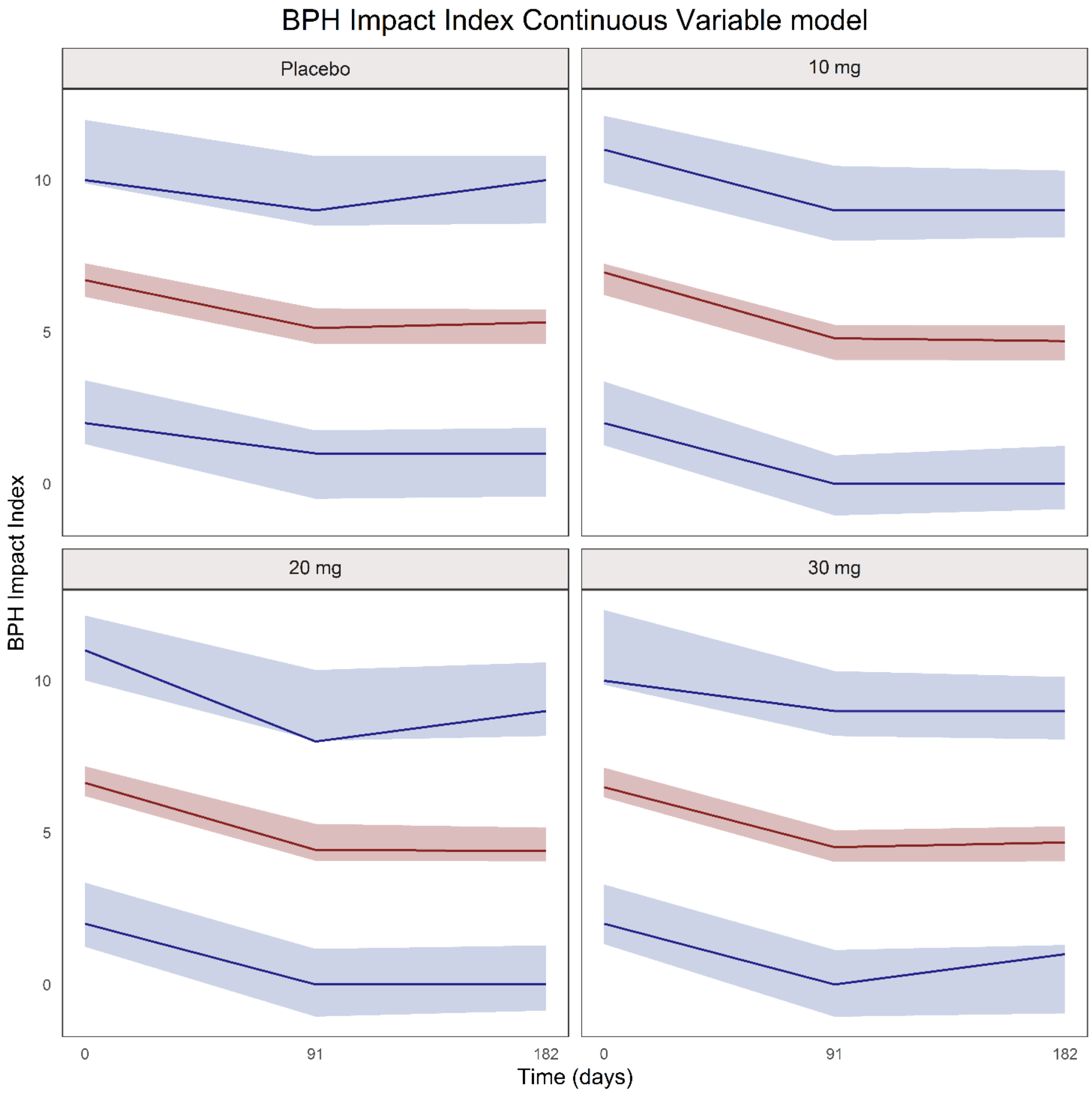

Figure S1.2.2 - Visual predictive check for the benign prostatic hyperplasia (BPH) impact index continuous variable model stratified by treatment arm comparing the median, 2.5th, and 97.5th percentiles of the observed data with the corresponding percentiles for simulated data displayed as 95% confidence intervals. Treatment effect was modeled as absent (placebo arm) or present (10 mg, 20 mg, and 30 mg degarelix arms). Reference model name: *BII-A*.

1.3 Ordered categorical models

1.3.1 Quality of Life score (*QoL-A*)

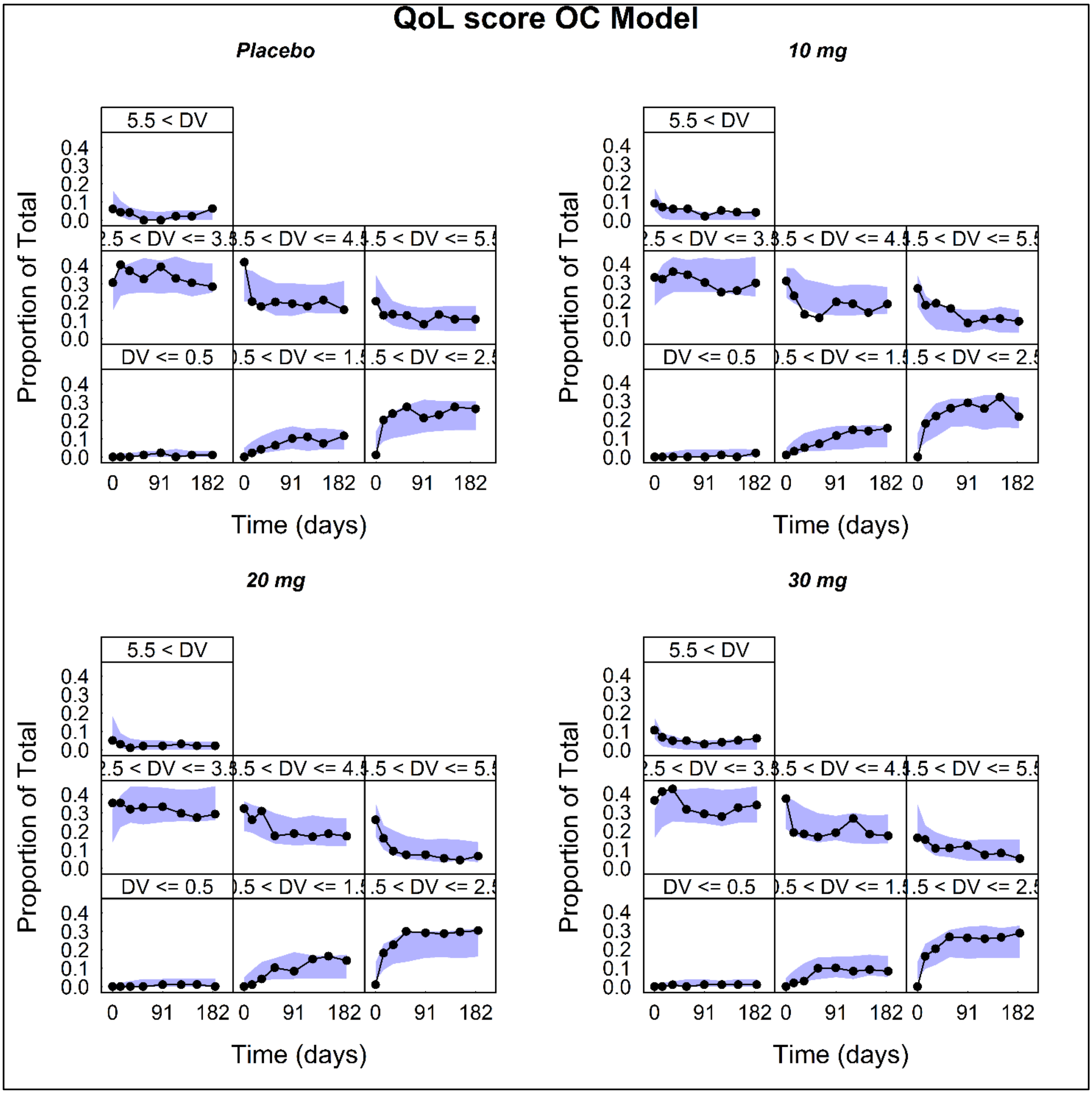

Figure S1.3.1 –Visual predictive check for the ordered categorical (OC) Quality of Life (QoL) score model stratified by treatment arm. The observed frequency of each score over time is shown as points and the shaded areas indicate the 95% confidence intervals of the frequencies of each score in 200 simulated datasets. DV: Dependent variable, i.e. observed score. Reference model name: *QoL-A*.

1.3.2. Benign Prostatic Hyperplasia Impact Index (*BII-B*)

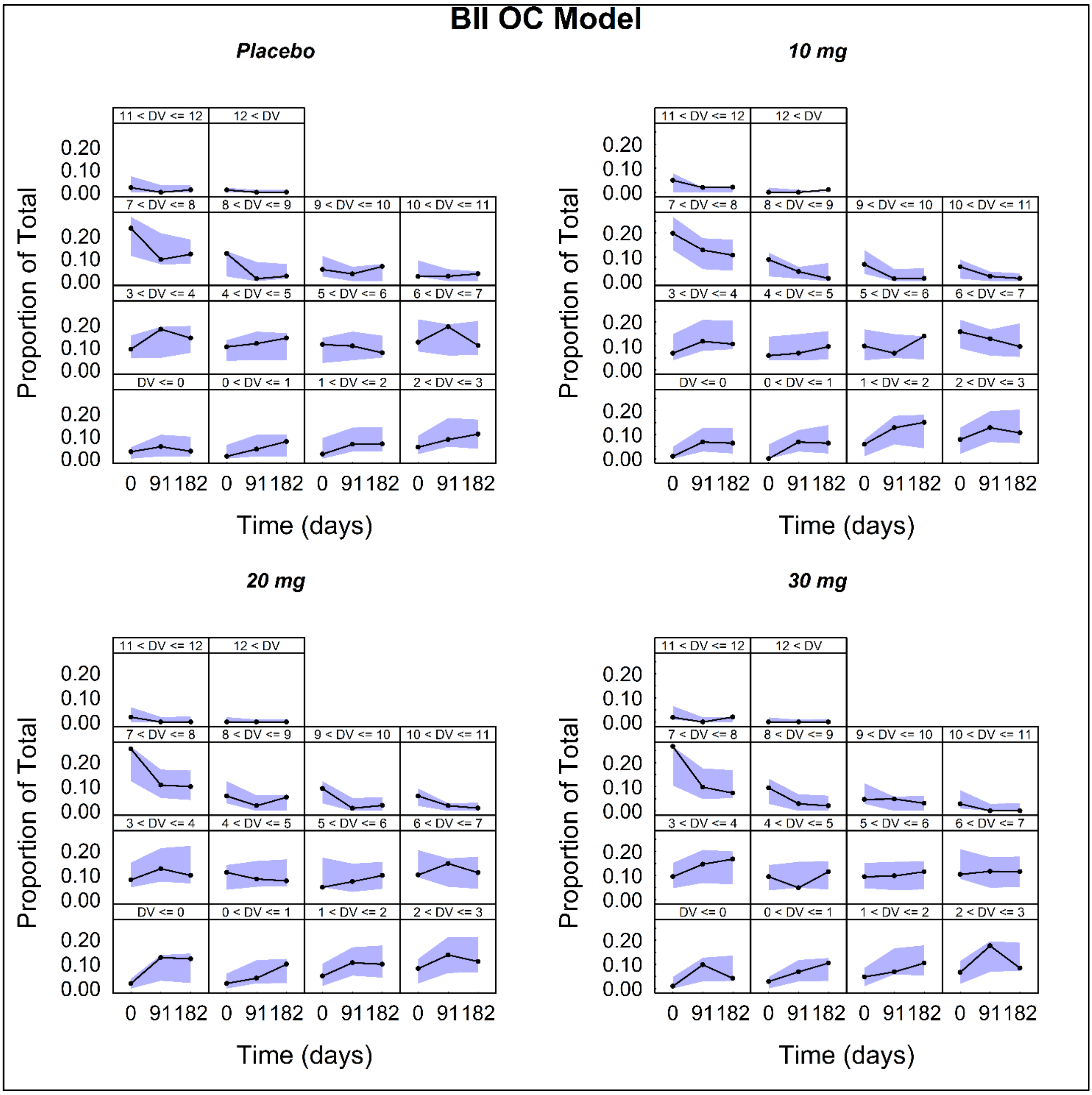

Figure S1.3.2 –Visual predictive check for the ordered categorical (OC) benign prostatic hyperplasia impact index (BII) model stratified by treatment arm. The observed frequency of each score over time is shown as points and the shaded areas indicate the 95% confidence intervals of the frequencies of each score in 200 simulated datasets. DV: Dependent variable, i.e. observed score. Reference model: *BII-B*.

1.4 Joint Bounded Integer Model

1.4.1 International Prostate Symptom Score

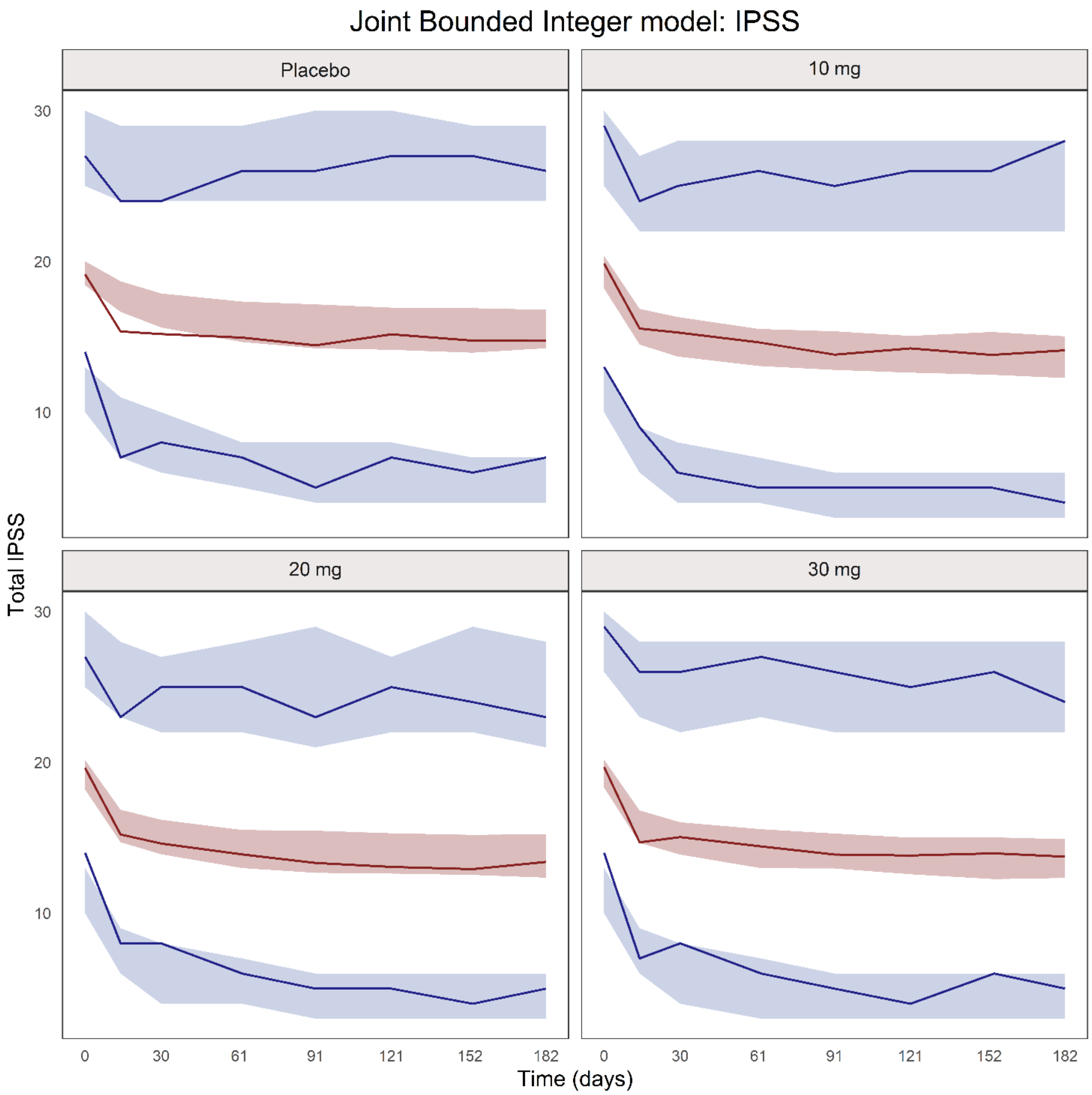

Figure S1.4.1 - Visual predictive check for the International Prostate Symptom Score (IPSS) in the joint bounded integer model stratified by treatment arm comparing the median, 2.5th, and 97.5th percentiles of the observed data with the corresponding percentiles for simulated data displayed as 95% confidence intervals. Treatment effect was modeled as absent (placebo arm) or present (10 mg, 20 mg, and 30 mg degarelix arms).

1.4.2 Quality of Life score

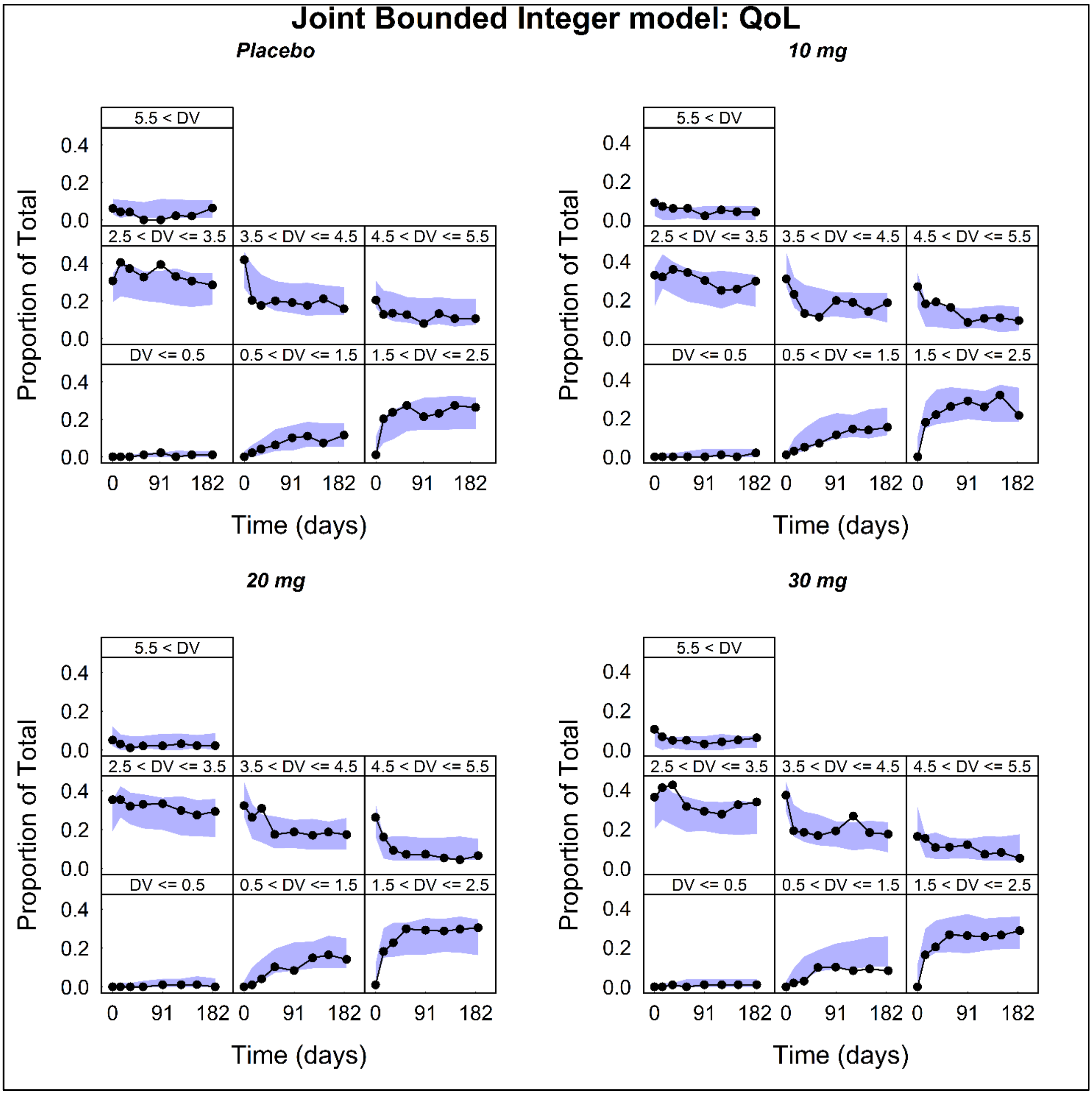

Figure S1.4.2 –Visual predictive check for the Quality of Life (QoL) score in the joint bounded integer model stratified by treatment arm. The observed frequency of each score over time is shown as points and the shaded areas indicate the 95% confidence intervals of the frequencies of each score in 200 simulated datasets. DV: Dependent variable, i.e. observed score.

1.4.3 Benign Prostatic Hyperplasia Impact Index

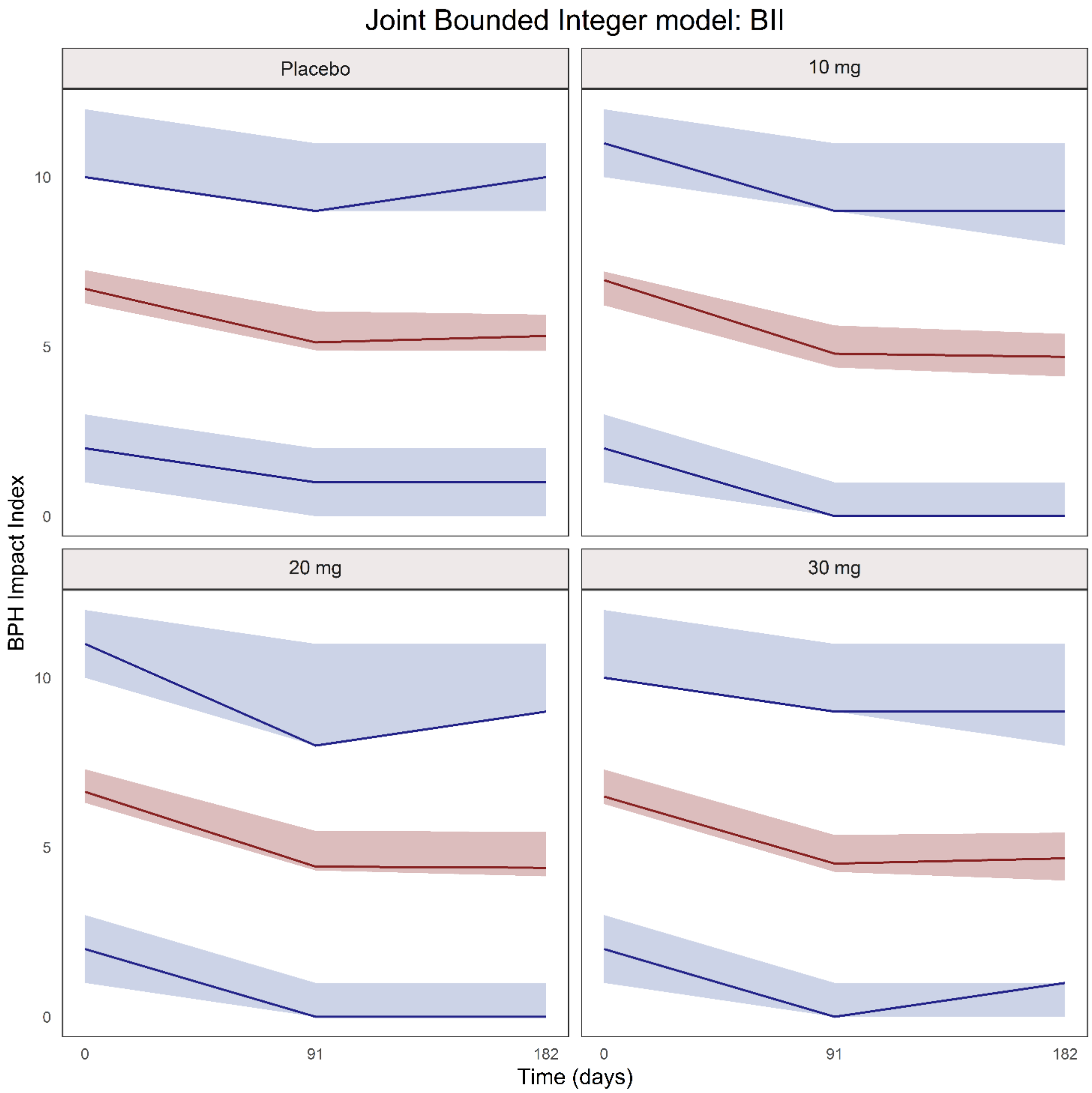

Figure S1.4.3 - Visual predictive check for the benign prostatic hyperplasia impact index (BII) in the joint bounded integer model stratified by treatment arm comparing the median, 2.5th, and 97.5th percentiles of the observed data with the corresponding percentiles for simulated data displayed as 95% confidence intervals. Treatment effect was modeled as absent (placebo arm) or present (10 mg, 20 mg, and 30 mg degarelix arms).

1.5 Exploratory Models

1.5.1 IPSS Continuous variable with random effect in residual variance

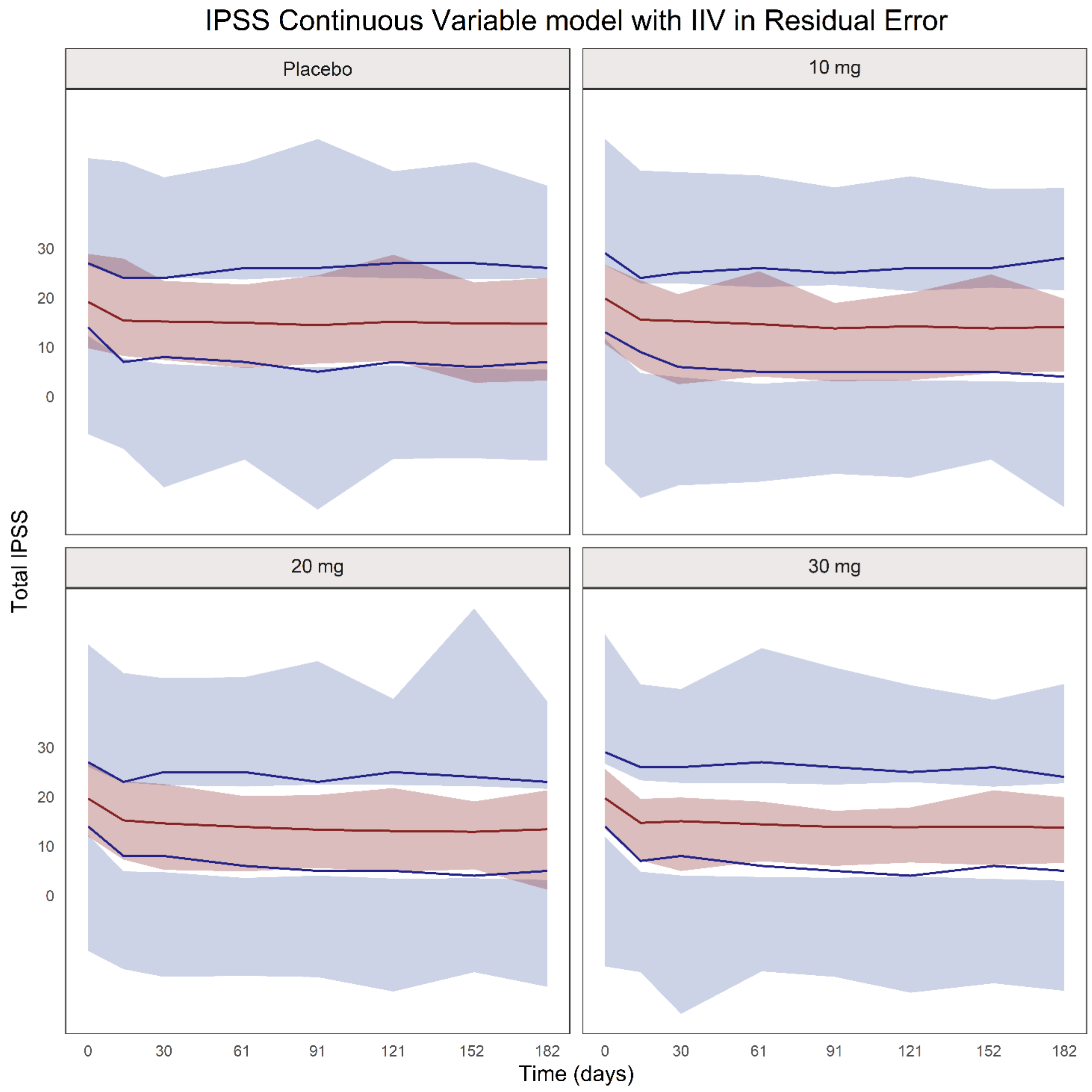

Figure S1.5 - Visual predictive check for the International Prostate Symptom Score (IPSS) continuous variable model with lognormally distributed inter-individual variability (IIV) in the additive residual error component. Stratification was performed by treatment arm, comparing the median, 2.5th, and 97.5th percentiles of the observed data with the corresponding percentiles for simulated data displayed as 95% confidence intervals. Treatment effect was modeled as absent (placebo arm) or present (10 mg, 20 mg, and 30 mg degarelix arms). Reference model name in Discussion set: *IPSS-A-2*.

2. Model parameter estimates

2.1 Joint Bounded Integer Model

Table 2.1 – Longitudinal parameter estimates in the joint bounded integer model, which simultaneously included patient responses to the International Prostate Symptom Score (IPSS), Quality of Life (QoL), and Benign Prostatic Hyperplasia Impact Index (BII) in the CS36 clinical trial. The IPSS cut-offs were used as the reference (fixed to calculated probit values) while the QoL and BII cut-offs were estimated. RSE: Relative standard error.

Parameter	Estimate	RSE
Baseline	0.13	13.3%
Asymptote	-0.298	8.9%
Progression half-life	22.3	9.8%
Drug effect	-0.142	14.4%
Standard deviation$_{\text{IPSS and QoL}}$ (g())	0.208	4.1%
Standard deviation$_{\text{BII}}$ (g())	0.469	5.7%
Interindividual variability		
Baseline	24.4%	5.7%
Asymptote	38.3%	5.9%
Progression half-life	74.6%	8.8%
Standard deviation$_{\text{IPSS and QoL}}$	60.5%	7.1%
Standard deviation$_{\text{BII}}$	30.7%	19%
Baseline – Standard deviation$_{\text{IPSS and QoL}}$ correlation	49.3%	6.5%

3. Model code

3.1 Bounded Integer Model for the International prostate Symptom Score (*IPSS-C*)

```
$SIZES     DIMNEW=20000 DIMTMP=900
$PROBLEM    BI model IPSS-C
$INPUT …
$DATA …
$PRED

BASE   = THETA(1) + ETA(1)
SD     = THETA(2)*EXP(ETA(5)) ; g() BI variance function
ASY = THETA(3) + ETA(2)
TPROG = THETA(4)*EXP(ETA(4))
SLPREL=ETA(3)

DRUG=0
IF(TIME.GT.0.AND.GRP.GT.0) DRUG=THETA(5); offset drug effect

IPRED  = BASE + ASY*(1-EXP(-LOG(2)/TPROG*TIME)) + SLPREL*TIME + DRUG

; --- Cutoffs determined as probits under a N(0,1) distribution ---

CO1 =  -1.91450583
CO2 =  -1.59321882
CO3 =  -1.38299413
CO4 =  -1.22064035
CO5 =  -1.08532491
CO6 =  -0.96742157
CO7 =  -0.86163412
CO8 =  -0.76470967
CO9 =  -0.67448975
CO10=  -0.58945580
CO11=  -0.50848806
CO12=  -0.43072730
CO13=  -0.35549042
CO14=  -0.28221615
CO15=  -0.21042839
CO16=  -0.13971030
CO17=  -0.06968492
CO18=   0.00000000
CO19=   0.06968492
CO20=   0.13971030
CO21=   0.21042839
CO22=   0.28221615
```

```
CO23=   0.35549042
CO24=   0.43072730
CO25=   0.50848806
CO26=   0.58945580
CO27=   0.67448975
CO28=   0.76470967
CO29=   0.86163412
CO30=   0.96742157
CO31=   1.08532491
CO32=   1.22064035
CO33=   1.38299413
CO34=   1.59321882
CO35=   1.91450583

; --- Probabilities computed ---

P0      =  PHI((CO1-IPRED)/SD)
P1      = PHI((CO2-IPRED)/SD)          -                    PHI((CO1-IPRED)/SD)
P2      =PHI((CO3-IPRED)/SD)           -                    PHI((CO2-IPRED)/SD)
P3      =PHI((CO4-IPRED)/SD)           -                    PHI((CO3-IPRED)/SD)
P4      =PHI((CO5-IPRED)/SD)           -                    PHI((CO4-IPRED)/SD)
P5      =PHI((CO6-IPRED)/SD)           -                    PHI((CO5-IPRED)/SD)
P6      =PHI((CO7-IPRED)/SD)           -                    PHI((CO6-IPRED)/SD)
P7      =PHI((CO8-IPRED)/SD)           -                    PHI((CO7-IPRED)/SD)
P8      =PHI((CO9-IPRED)/SD)           -                    PHI((CO8-IPRED)/SD)
P9      =PHI((CO10-IPRED)/SD) -     PHI((CO9-IPRED)/SD)
P10     =PHI((CO11-IPRED)/SD) -     PHI((CO10-IPRED)/SD)
P11     =   PHI((CO12-IPRED)/SD) -   PHI((CO11-IPRED)/SD)
P12     =   PHI((CO13-IPRED)/SD) -   PHI((CO12-IPRED)/SD)
P13     =   PHI((CO14-IPRED)/SD) -   PHI((CO13-IPRED)/SD)
P14     =   PHI((CO15-IPRED)/SD) -   PHI((CO14-IPRED)/SD)
P15     =   PHI((CO16-IPRED)/SD) -   PHI((CO15-IPRED)/SD)
P16     =   PHI((CO17-IPRED)/SD) -   PHI((CO16-IPRED)/SD)
P17     =   PHI((CO18-IPRED)/SD) -   PHI((CO17-IPRED)/SD)
P18     =   PHI((CO19-IPRED)/SD) -   PHI((CO18-IPRED)/SD)
P19     =   PHI((CO20-IPRED)/SD) -   PHI((CO19-IPRED)/SD)
P20     =   PHI((CO21-IPRED)/SD) -   PHI((CO20-IPRED)/SD)
P21     =   PHI((CO22-IPRED)/SD) -   PHI((CO21-IPRED)/SD)
P22     =   PHI((CO23-IPRED)/SD) -   PHI((CO22-IPRED)/SD)
P23     =   PHI((CO24-IPRED)/SD) -   PHI((CO23-IPRED)/SD)
P24     =   PHI((CO25-IPRED)/SD) -   PHI((CO24-IPRED)/SD)
P25     =   PHI((CO26-IPRED)/SD) -   PHI((CO25-IPRED)/SD)
P26     =   PHI((CO27-IPRED)/SD) -   PHI((CO26-IPRED)/SD)
P27     =   PHI((CO28-IPRED)/SD) -   PHI((CO27-IPRED)/SD)
P28     =   PHI((CO29-IPRED)/SD) -   PHI((CO28-IPRED)/SD)
P29     =   PHI((CO30-IPRED)/SD) -   PHI((CO29-IPRED)/SD)
```

```
P30   =   PHI((CO31-IPRED)/SD) -    PHI((CO30-IPRED)/SD)
P31   =   PHI((CO32-IPRED)/SD) -    PHI((CO31-IPRED)/SD)
P32   =   PHI((CO33-IPRED)/SD) -    PHI((CO32-IPRED)/SD)
P33   =   PHI((CO34-IPRED)/SD) -    PHI((CO33-IPRED)/SD)
P34   =   PHI((CO35-IPRED)/SD) -    PHI((CO34-IPRED)/SD)
P35   =   1 -PHI((CO35-IPRED)/SD)

; --- Set probabilities ---
IF(DV.GE.0) Y1 = P0
IF(DV.GE.1) Y1 = P1
IF(DV.GE.2) Y1 = P2
IF(DV.GE.3) Y1 = P3
IF(DV.GE.4) Y1 = P4
IF(DV.GE.5) Y1 = P5
IF(DV.GE.6) Y1 = P6
IF(DV.GE.7) Y1 = P7
IF(DV.GE.8) Y1 = P8
IF(DV.GE.9) Y1 = P9
IF(DV.GE.10) Y1 = P10
IF(DV.GE.11) Y1 = P11
IF(DV.GE.12) Y1 = P12
IF(DV.GE.13) Y1 = P13
IF(DV.GE.14) Y1 = P14
IF(DV.GE.15) Y1 = P15
IF(DV.GE.16) Y1 = P16
IF(DV.GE.17) Y1 = P17
IF(DV.GE.18) Y1 = P18
IF(DV.GE.19) Y1 = P19
IF(DV.GE.20) Y1 = P20
IF(DV.GE.21) Y1 = P21
IF(DV.GE.22) Y1 = P22
IF(DV.GE.23) Y1 = P23
IF(DV.GE.24) Y1 = P24
IF(DV.GE.25) Y1 = P25
IF(DV.GE.26) Y1 = P26
IF(DV.GE.27) Y1 = P27
IF(DV.GE.28) Y1 = P28
IF(DV.GE.29) Y1 = P29
IF(DV.GE.30) Y1 = P30
IF(DV.GE.31) Y1 = P31
IF(DV.GE.32) Y1 = P32
IF(DV.GE.33) Y1 = P33
IF(DV.GE.34) Y1 = P34
IF(DV.GE.35) Y1 = P35

Y=Y1
```

```
; --- Cumulative probabilities ---
CUP0   =  P0
CUP1 =              CUP0  +      P1
CUP2 =              CUP1  +      P2
CUP3 =              CUP2  +      P3
CUP4 =              CUP3  +      P4
CUP5 =              CUP4  +      P5
CUP6 =              CUP5  +      P6
CUP7 =              CUP6  +      P7
CUP8 =              CUP7  +      P8
CUP9 =              CUP8  +      P9
CUP10               =            CUP9   +  P10
CUP11               =            CUP10  +  P11
CUP12               =            CUP11  +  P12
CUP13               =            CUP12  +  P13
CUP14               =            CUP13  +  P14
CUP15               =            CUP14  +  P15
CUP16               =            CUP15  +  P16
CUP17               =            CUP16  +  P17
CUP18               =            CUP17  +  P18
CUP19               =            CUP18  +  P19
CUP20               =            CUP19  +  P20
CUP21               =            CUP20  +  P21
CUP22               =            CUP21  +  P22
CUP23               =            CUP22  +  P23
CUP24               =            CUP23  +  P24
CUP25               =            CUP24  +  P25
CUP26               =            CUP25  +  P26
CUP27               =            CUP26  +  P27
CUP28               =            CUP27  +  P28
CUP29               =            CUP28  +  P29
CUP30               =            CUP29  +  P30
CUP31               =            CUP30  +  P31
CUP32               =            CUP31  +  P32
CUP33               =            CUP32  +  P33
CUP34               =            CUP33  +  P34
CUP35               =            1

; Code to generate VPC using flip_comments in PsN
;Sim_start
;  ;Simulations
;   IF(ICALL.EQ.4) THEN
;     CALL RANDOM (2,R)
;     IF(R.LE.   CUP0)                 DV= 0
;     IF(R.GT.CUP0.AND.R.LE.CUP1)   DV=            1
```

```
;    IF(R.GT.CUP1.AND.R.LE.CUP2)   DV=      2
;    IF(R.GT.CUP2.AND.R.LE.CUP3)   DV=      3
;    IF(R.GT.CUP3.AND.R.LE.CUP4)   DV=      4
;    IF(R.GT.CUP4.AND.R.LE.CUP5)   DV=      5
;    IF(R.GT.CUP5.AND.R.LE.CUP6)   DV=      6
;    IF(R.GT.CUP6.AND.R.LE.CUP7)   DV=      7
;    IF(R.GT.CUP7.AND.R.LE.CUP8)   DV=      8
;    IF(R.GT.CUP8.AND.R.LE.CUP9)   DV=      9
;    IF(R.GT.CUP9.AND.R.LE.CUP10  )   DV=   10
;    IF(R.GT.CUP10.AND.R.LE.CUP11 )   DV=   11
;    IF(R.GT.CUP11.AND.R.LE.CUP12 )   DV=   12
;    IF(R.GT.CUP12.AND.R.LE.CUP13 )   DV=   13
;    IF(R.GT.CUP13.AND.R.LE.CUP14 )   DV=   14
;    IF(R.GT.CUP14.AND.R.LE.CUP15 )   DV=   15
;    IF(R.GT.     CUP15  .AND.R.LE.     CUP16     )  DV=     16
;    IF(R.GT.     CUP16  .AND.R.LE.     CUP17     )  DV=     17
;    IF(R.GT.     CUP17  .AND.R.LE.     CUP18     )  DV=     18
;    IF(R.GT.     CUP18  .AND.R.LE.     CUP19     )  DV=     19
;    IF(R.GT.     CUP19  .AND.R.LE.     CUP20     )  DV=     20
;    IF(R.GT.     CUP20  .AND.R.LE.     CUP21     )  DV=     21
;    IF(R.GT.     CUP21  .AND.R.LE.     CUP22     )  DV=     22
;    IF(R.GT.     CUP22  .AND.R.LE.     CUP23     )  DV=     23
;    IF(R.GT.     CUP23  .AND.R.LE.     CUP24     )  DV=     24
;    IF(R.GT.     CUP24  .AND.R.LE.     CUP25     )  DV=     25
;    IF(R.GT.     CUP25  .AND.R.LE.     CUP26     )  DV=     26
;    IF(R.GT.     CUP26  .AND.R.LE.     CUP27     )  DV=     27
;    IF(R.GT.     CUP27  .AND.R.LE.     CUP28     )  DV=     28
;    IF(R.GT.     CUP28  .AND.R.LE.     CUP29     )  DV=     29
;    IF(R.GT.     CUP29  .AND.R.LE.     CUP30     )  DV=     30
;    IF(R.GT.     CUP30  .AND.R.LE.     CUP31     )  DV=     31
;    IF(R.GT.     CUP31  .AND.R.LE.     CUP32     )  DV=     32
;    IF(R.GT.     CUP32  .AND.R.LE.     CUP33     )  DV=     33
;    IF(R.GT.     CUP33  .AND.R.LE.     CUP34     )  DV=     34
;    IF(R.GT.     CUP34  .AND.R.LE.     CUP35     )  DV=     35
;   ENDIF
;Sim_end

; Initial estimates

$THETA
(0.108) ; 1 BASE
(0, 0.163) ; 2 SD
(-0.29) ; 3 ASY
(0, 17.8) ; 4 TPROG
(-0.13) ; DRUG
```

```
$OMEGA
 0.0777 ; IIV_BASE
$OMEGA BLOCK(2)
 0.12  ;  IIV_ASY
 0.000084 0.0001  ; SLPREL – fixing this parameter to 0 yields BI model IPSS-D
$OMEGA
 0.518 ; IIV_TPROG
$OMEGA
 0.406 ; g() function IIV parameter – fixing this parameter yields model IPSS-B

; below code also necessary to allow VPC generation using flip_comments in PsN
;Sim_start
;$SIMULATION (54321) (18877 UNIFORM) ONLYSIM NOPREDICTION NSUB=1

$ESTIMATION MAXEVAL=9999 METHOD=1 LAPLACE LIKE PRINT=1
$COV PRINT=E
;Sim_end
```

3.2 Joint Bounded Integer Model

```
$SIZES DIMNEW=20000 DIMTMP=900
$PROBLEM Joint BI Model  ; IPSS & QoL & BII
$INPUT ...

; FLAG=1 TOTAL IPSS
; FLAG=4 QOL SCORE
; FLAG=5 BPH-II SCORE

$DATA cs36data.csv IGNORE=(FLAG.EQ.2) IGNORE=(FLAG.EQ.3) IGNORE=@

$PRED
BASE   = THETA(1) + ETA(1)
IF(FLAG.LT.5) SD = THETA(2) * EXP(ETA(2))    ; g() function for IPSS and QoL
IF(FLAG.EQ.5) SD = THETA(25) * EXP(ETA(5))   ; g() function for BII
ASY = THETA(3) + ETA(3)
TPROG = THETA(4)*EXP(ETA(4))

DRUG=0
IF(TIME.GT.0.AND.GRP.GT.0) DRUG=THETA(5)

IPRED  = BASE + ASY*(1-EXP(-LOG(2)/TPROG*TIME)) + DRUG

; --- Cutoffs ---

; IPSS
IF(FLAG.EQ.1) THEN
 CO1 =  -1.91450583
 CO2 =  -1.59321882
 CO3 =  -1.38299413
 CO4 =  -1.22064035
 CO5 =  -1.08532491
 CO6 =  -0.96742157
 CO7 =  -0.86163412
 CO8 =  -0.76470967
 CO9 =  -0.67448975
 CO10=  -0.58945580
 CO11=  -0.50848806
 CO12=  -0.43072730
 CO13=  -0.35549042
 CO14=  -0.28221615
 CO15=  -0.21042839
 CO16=  -0.13971030
 CO17=  -0.06968492
 CO18=   0.00000000
```

```
 CO19=   0.06968492
 CO20=   0.13971030
 CO21=   0.21042839
 CO22=   0.28221615
 CO23=   0.35549042
 CO24=   0.43072730
 CO25=   0.50848806
 CO26=   0.58945580
 CO27=   0.67448975
 CO28=   0.76470967
 CO29=   0.86163412
 CO30=   0.96742157
 CO31=   1.08532491
 CO32=   1.22064035
 CO33=   1.38299413
 CO34=   1.59321882
 CO35=   1.91450583
ENDIF

; Estimate QoL cut-offs
IF(FLAG.EQ.4) THEN
 CO1 = THETA(6)
 CO2 = CO1 + THETA(7)
 CO3 = CO2 + THETA(8)
 CO4 = CO3 + THETA(9)
 CO5 = CO4 + THETA(10)
 CO6 = CO5 + THETA(11)
ENDIF

; Estimate BII cut-offs
IF(FLAG.EQ.5) THEN
 CO1 = THETA(12)
 CO2 = CO1 + THETA(13)
 CO3 = CO2 + THETA(14)
 CO4 = CO3 + THETA(15)
 CO5 = CO4 + THETA(16)
 CO6 = CO5 + THETA(17)
 CO7 = CO6 + THETA(18)
 CO8 = CO7 + THETA(19)
 CO9 = CO8 + THETA(20)
 CO10 = CO9 + THETA(21)
 CO11 = CO10 + THETA(22)
 CO12 = CO11 + THETA(23)
 CO13 = CO12 + THETA(24)
ENDIF
```

```
; --- Probabilities computed IPSS ---
IF(FLAG.EQ.1) THEN
 P0   =           PHI((CO1-IPRED)/SD)
 P1   =           PHI((CO2-IPRED)/SD)     -           PHI((CO1-IPRED)/SD)
 P2   =           PHI((CO3-IPRED)/SD)     -           PHI((CO2-IPRED)/SD)
 P3   =           PHI((CO4-IPRED)/SD)     -           PHI((CO3-IPRED)/SD)
 P4   =           PHI((CO5-IPRED)/SD)     -           PHI((CO4-IPRED)/SD)
 P5   =           PHI((CO6-IPRED)/SD)     -           PHI((CO5-IPRED)/SD)
 P6   =           PHI((CO7-IPRED)/SD)     -           PHI((CO6-IPRED)/SD)
 P7   =           PHI((CO8-IPRED)/SD)     -           PHI((CO7-IPRED)/SD)
 P8   =           PHI((CO9-IPRED)/SD)     -           PHI((CO8-IPRED)/SD)
 P9   =           PHI((CO10-IPRED)/SD) -    PHI((CO9-IPRED)/SD)
 P10  =           PHI((CO11-IPRED)/SD) -    PHI((CO10-IPRED)/SD)
 P11  = PHI((CO12-IPRED)/SD) -  PHI((CO11-IPRED)/SD)
 P12  = PHI((CO13-IPRED)/SD) -  PHI((CO12-IPRED)/SD)
 P13  = PHI((CO14-IPRED)/SD) -  PHI((CO13-IPRED)/SD)
 P14  = PHI((CO15-IPRED)/SD) -  PHI((CO14-IPRED)/SD)
 P15  = PHI((CO16-IPRED)/SD) -  PHI((CO15-IPRED)/SD)
 P16  = PHI((CO17-IPRED)/SD) -  PHI((CO16-IPRED)/SD)
 P17  = PHI((CO18-IPRED)/SD) -  PHI((CO17-IPRED)/SD)
 P18  = PHI((CO19-IPRED)/SD) -  PHI((CO18-IPRED)/SD)
 P19  = PHI((CO20-IPRED)/SD) -  PHI((CO19-IPRED)/SD)
 P20  = PHI((CO21-IPRED)/SD) -  PHI((CO20-IPRED)/SD)
 P21  = PHI((CO22-IPRED)/SD) -  PHI((CO21-IPRED)/SD)
 P22  = PHI((CO23-IPRED)/SD) -  PHI((CO22-IPRED)/SD)
 P23  = PHI((CO24-IPRED)/SD) -  PHI((CO23-IPRED)/SD)
 P24  = PHI((CO25-IPRED)/SD) -  PHI((CO24-IPRED)/SD)
 P25  = PHI((CO26-IPRED)/SD) -  PHI((CO25-IPRED)/SD)
 P26  = PHI((CO27-IPRED)/SD) -  PHI((CO26-IPRED)/SD)
 P27  = PHI((CO28-IPRED)/SD) -  PHI((CO27-IPRED)/SD)
 P28  = PHI((CO29-IPRED)/SD) -  PHI((CO28-IPRED)/SD)
 P29  = PHI((CO30-IPRED)/SD) -  PHI((CO29-IPRED)/SD)
 P30  = PHI((CO31-IPRED)/SD) -  PHI((CO30-IPRED)/SD)
 P31  = PHI((CO32-IPRED)/SD) -  PHI((CO31-IPRED)/SD)
 P32  = PHI((CO33-IPRED)/SD) -  PHI((CO32-IPRED)/SD)
 P33  = PHI((CO34-IPRED)/SD) -  PHI((CO33-IPRED)/SD)
 P34  = PHI((CO35-IPRED)/SD) -  PHI((CO34-IPRED)/SD)
 P35  = 1 -       PHI((CO35-IPRED)/SD)
ENDIF

; --- Probabilities computed QoL ---
IF(FLAG.EQ.4) THEN
 P0   =           PHI((CO1-IPRED)/SD)
 P1   =           PHI((CO2-IPRED)/SD)     -           PHI((CO1-IPRED)/SD)
 P2   =           PHI((CO3-IPRED)/SD)     -           PHI((CO2-IPRED)/SD)
 P3   =           PHI((CO4-IPRED)/SD)     -           PHI((CO3-IPRED)/SD)
```

```
 P4   =       PHI((CO5-IPRED)/SD)      -       PHI((CO4-IPRED)/SD)
 P5   =       PHI((CO6-IPRED)/SD)      -       PHI((CO5-IPRED)/SD)
 P6   =       1 -     PHI((CO6-IPRED)/SD)
ENDIF

; --- Probabilities computed BII ---
IF(FLAG.EQ.5) THEN
 P0   =       PHI((CO1-IPRED)/SD)
 P1   =       PHI((CO2-IPRED)/SD)      -       PHI((CO1-IPRED)/SD)
 P2   =       PHI((CO3-IPRED)/SD)      -       PHI((CO2-IPRED)/SD)
 P3   =       PHI((CO4-IPRED)/SD)      -       PHI((CO3-IPRED)/SD)
 P4   =       PHI((CO5-IPRED)/SD)      -       PHI((CO4-IPRED)/SD)
 P5   =       PHI((CO6-IPRED)/SD)      -       PHI((CO5-IPRED)/SD)
 P6   =       PHI((CO7-IPRED)/SD)      -       PHI((CO6-IPRED)/SD)
 P7   =       PHI((CO8-IPRED)/SD)      -       PHI((CO7-IPRED)/SD)
 P8   =       PHI((CO9-IPRED)/SD)      -       PHI((CO8-IPRED)/SD)
 P9   =       PHI((CO10-IPRED)/SD) -   PHI((CO9-IPRED)/SD)
 P10  =       PHI((CO11-IPRED)/SD) -   PHI((CO10-IPRED)/SD)
 P11  =  PHI((CO12-IPRED)/SD) -  PHI((CO11-IPRED)/SD)
 P12  =  PHI((CO13-IPRED)/SD) -  PHI((CO12-IPRED)/SD)
 P13  =  1 -      PHI((CO13-IPRED)/SD)
ENDIF

; --- Set probabilities ---
IF(DV.GE.0) Y1 = P0
IF(DV.GE.1) Y1 = P1
IF(DV.GE.2) Y1 = P2
IF(DV.GE.3) Y1 = P3
IF(DV.GE.4) Y1 = P4
IF(DV.GE.5) Y1 = P5
IF(DV.GE.6) Y1 = P6
IF(DV.GE.7) Y1 = P7
IF(DV.GE.8) Y1 = P8
IF(DV.GE.9) Y1 = P9
IF(DV.GE.10) Y1 = P10
IF(DV.GE.11) Y1 = P11
IF(DV.GE.12) Y1 = P12
IF(DV.GE.13) Y1 = P13
IF(DV.GE.14) Y1 = P14
IF(DV.GE.15) Y1 = P15
IF(DV.GE.16) Y1 = P16
IF(DV.GE.17) Y1 = P17
IF(DV.GE.18) Y1 = P18
IF(DV.GE.19) Y1 = P19
IF(DV.GE.20) Y1 = P20
IF(DV.GE.21) Y1 = P21
```

```
IF(DV.GE.22) Y1 = P22
IF(DV.GE.23) Y1 = P23
IF(DV.GE.24) Y1 = P24
IF(DV.GE.25) Y1 = P25
IF(DV.GE.26) Y1 = P26
IF(DV.GE.27) Y1 = P27
IF(DV.GE.28) Y1 = P28
IF(DV.GE.29) Y1 = P29
IF(DV.GE.30) Y1 = P30
IF(DV.GE.31) Y1 = P31
IF(DV.GE.32) Y1 = P32
IF(DV.GE.33) Y1 = P33
IF(DV.GE.34) Y1 = P34
IF(DV.GE.35) Y1 = P35

Y=Y1


; --- Cumulative probabilities ---

; IPSS
IF(FLAG.EQ.1) THEN
 CUP0   =   P0
 CUP1 =          CUP0  +     P1
 CUP2 =          CUP1  +     P2
 CUP3 =          CUP2  +     P3
 CUP4 =          CUP3  +     P4
 CUP5 =          CUP4  +     P5
 CUP6 =          CUP5  +     P6
 CUP7 =          CUP6  +     P7
 CUP8 =          CUP7  +     P8
 CUP9 =          CUP8  +     P9
 CUP10=          CUP9  +  P10
 CUP11=          CUP10  +  P11
 CUP12=          CUP11  +  P12
 CUP13=          CUP12  +  P13
 CUP14=          CUP13  +  P14
 CUP15=          CUP14  +  P15
 CUP16=          CUP15  +  P16
 CUP17=          CUP16  +  P17
 CUP18=          CUP17  +  P18
 CUP19=          CUP18  +  P19
 CUP20=          CUP19  +  P20
 CUP21=          CUP20  +  P21
 CUP22=          CUP21  +  P22
 CUP23=          CUP22  +  P23
```

```
 CUP24=              CUP23  +  P24
 CUP25=              CUP24  +  P25
 CUP26=              CUP25  +  P26
 CUP27=              CUP26  +  P27
 CUP28=              CUP27  +  P28
 CUP29=              CUP28  +  P29
 CUP30=              CUP29  +  P30
 CUP31=              CUP30  +  P31
 CUP32=              CUP31  +  P32
 CUP33=              CUP32  +  P33
 CUP34=              CUP33  +  P34
 CUP35=              1
ENDIF

; QoL
IF(FLAG.EQ.4) THEN
 CUP0   =   P0
 CUP1 = CUP0   +      P1
 CUP2 =CUP1   +       P2
 CUP3 =CUP2   +       P3
 CUP4 =CUP3   +       P4
 CUP5 =CUP4   +       P5
 CUP6 =1
ENDIF

IF(FLAG.EQ.5) THEN
 CUP0   =   P0
 CUP1 =CUP0   +       P1
 CUP2 =CUP1   +       P2
 CUP3 =CUP2   +       P3
 CUP4 =CUP3   +       P4
 CUP5 =CUP4   +       P5
 CUP6 =CUP5   +       P6
 CUP7 =CUP6   +       P7
 CUP8 =CUP7   +       P8
 CUP9 =CUP8   +       P9
 CUP10=              CUP9   +  P10
 CUP11=              CUP10  +  P11
 CUP12=              CUP11  +  P12
 CUP13=              1
ENDIF


;Sim_start
;  ;Simulations
;   IF(ICALL.EQ.4) THEN
```

```
;     IF(FLAG.EQ.1) THEN
;      CALL RANDOM (2,R)
;      IF(R.LE.   CUP0)                     DV= 0
;      IF(R.GT.             CUP0             .AND.R.LE.       CUP1              )  DV=            1
;      IF(R.GT.             CUP1             .AND.R.LE.       CUP2              )  DV=            2
;      IF(R.GT.             CUP2             .AND.R.LE.       CUP3              )  DV=            3
;      IF(R.GT.             CUP3             .AND.R.LE.       CUP4              )  DV=            4
;      IF(R.GT.             CUP4             .AND.R.LE.       CUP5              )  DV=            5
;      IF(R.GT.             CUP5             .AND.R.LE.       CUP6              )  DV=            6
;      IF(R.GT.             CUP6             .AND.R.LE.       CUP7              )  DV=            7
;      IF(R.GT.             CUP7             .AND.R.LE.       CUP8              )  DV=            8
;      IF(R.GT.             CUP8             .AND.R.LE.       CUP9              )  DV=            9
;      IF(R.GT.             CUP9             .AND.R.LE.       CUP10             )  DV=            10
;      IF(R.GT.             CUP10   .AND.R.LE.                CUP11             )  DV=            11
;      IF(R.GT.             CUP11   .AND.R.LE.                CUP12             )  DV=            12
;      IF(R.GT.             CUP12   .AND.R.LE.                CUP13             )  DV=            13
;      IF(R.GT.             CUP13   .AND.R.LE.                CUP14             )  DV=            14
;      IF(R.GT.             CUP14   .AND.R.LE.                CUP15             )  DV=            15
;      IF(R.GT.             CUP15   .AND.R.LE.                CUP16             )  DV=            16
;      IF(R.GT.             CUP16   .AND.R.LE.                CUP17             )  DV=            17
;      IF(R.GT.             CUP17   .AND.R.LE.                CUP18             )  DV=            18
;      IF(R.GT.             CUP18   .AND.R.LE.                CUP19             )  DV=            19
;      IF(R.GT.             CUP19   .AND.R.LE.                CUP20             )  DV=            20
;      IF(R.GT.             CUP20   .AND.R.LE.                CUP21             )  DV=            21
;      IF(R.GT.             CUP21   .AND.R.LE.                CUP22             )  DV=            22
;      IF(R.GT.             CUP22   .AND.R.LE.                CUP23             )  DV=            23
;      IF(R.GT.             CUP23   .AND.R.LE.                CUP24             )  DV=            24
;      IF(R.GT.             CUP24   .AND.R.LE.                CUP25             )  DV=            25
;      IF(R.GT.             CUP25   .AND.R.LE.                CUP26             )  DV=            26
;      IF(R.GT.             CUP26   .AND.R.LE.                CUP27             )  DV=            27
;      IF(R.GT.             CUP27   .AND.R.LE.                CUP28             )  DV=            28
;      IF(R.GT.             CUP28   .AND.R.LE.                CUP29             )  DV=            29
;      IF(R.GT.             CUP29   .AND.R.LE.                CUP30             )  DV=            30
;      IF(R.GT.             CUP30   .AND.R.LE.                CUP31             )  DV=            31
;      IF(R.GT.             CUP31   .AND.R.LE.                CUP32             )  DV=            32
;      IF(R.GT.             CUP32   .AND.R.LE.                CUP33             )  DV=            33
;      IF(R.GT.             CUP33   .AND.R.LE.                CUP34             )  DV=            34
;      IF(R.GT.             CUP34   .AND.R.LE.                CUP35             )  DV=            35
;     ENDIF
;            IF(FLAG.EQ.4) THEN
;      CALL RANDOM (2,R)
;      IF(R.LE.   CUP0)                      DV= 0
;      IF(R.GT.             CUP0             .AND.R.LE.       CUP1              )  DV=            1
;      IF(R.GT.             CUP1             .AND.R.LE.       CUP2              )  DV=            2
;      IF(R.GT.             CUP2             .AND.R.LE.       CUP3              )  DV=            3
;      IF(R.GT.             CUP3             .AND.R.LE.       CUP4              )  DV=            4
```

```
;     IF(R.GT.            CUP4            .AND.R.LE.      CUP5            )  DV=          5
;     IF(R.GT.            CUP5            .AND.R.LE.      CUP6            )  DV=          6
;    ENDIF
;    IF(FLAG.EQ.5) THEN
;     CALL RANDOM (2,R)
;     IF(R.LE.   CUP0)                  DV= 0
;     IF(R.GT.            CUP0            .AND.R.LE.      CUP1            )  DV=          1
;     IF(R.GT.            CUP1            .AND.R.LE.      CUP2            )  DV=          2
;     IF(R.GT.            CUP2            .AND.R.LE.      CUP3            )  DV=          3
;     IF(R.GT.            CUP3            .AND.R.LE.      CUP4            )  DV=          4
;     IF(R.GT.            CUP4            .AND.R.LE.      CUP5            )  DV=          5
;     IF(R.GT.            CUP5            .AND.R.LE.      CUP6            )  DV=          6
;     IF(R.GT.            CUP6            .AND.R.LE.      CUP7            )  DV=          7
;     IF(R.GT.            CUP7            .AND.R.LE.      CUP8            )  DV=          8
;     IF(R.GT.            CUP8            .AND.R.LE.      CUP9            )  DV=          9
;     IF(R.GT.            CUP9            .AND.R.LE.      CUP10           )  DV=          10
;     IF(R.GT.            CUP10   .AND.R.LE.              CUP11           )  DV=          11
;     IF(R.GT.            CUP11   .AND.R.LE.              CUP12           )  DV=          12
;     IF(R.GT.            CUP12   .AND.R.LE.              CUP13           )  DV=          13
;    ENDIF
; ENDIF
;Sim_end

$THETA
(0.11) ; 1 BASE
(0.207) ; 2 SD
(-0.286) ; 3 ASY
(0, 21.7) ; 4 TPROG
(-0.133) ; DRUG
(-1.42) ; QOL CO1
(0, 0.656) ; QOL DELTACO2
(0, 0.391) ; QOL DELTACO3
(0, 0.335) ; QOL DELTACO4
(0, 0.309) ; QOL DELTACO5
(0, 0.401) ; QOL DELTACO6
(-1.21) ; BII CO1
(0, 0.262) ; BII DELTACO2
(0, 0.244) ; BII DELTACO3
(0, 0.21) ; BII DELTACO4
(0, 0.195) ; BII DELTACO5
(0, 0.138) ; BII DELTACO6
(0, 0.139) ; BII DELTACO7
(0, 0.199) ; BII DELTACO8
(0, 0.327) ; BII DELTACO9
(0, 0.181) ; BII DELTACO10
(0, 0.248) ; BII DELTACO11
```

```
(0, 0.285) ; BII DELTACO12
(0, 0.722) ; BII DELTACO13
(0, 0.456) ; BII SD

$OMEGA BLOCK(2)
0.06 ; 1 IIV_BASE
0.1 0.37 ; 2 IIV_SD
$OMEGA 0.143 ; 3 IIV_ASY
$OMEGA 0.528 ; 4 IIV_TPROG
$OMEGA 0.0952 ; 5 IIV_SD-BII

;Sim_start
;$SIMULATION (54321) (18877 UNIFORM) ONLYSIM NOPREDICTION NSUB=1

$ESTIMATION MAXEVAL=9999 METHOD=1 LAPLACE LIKE PRINT=1 MCETA=10
$COV PRINT=E

;Sim_end
```
